# FMRP activity and control of Csw/SHP2 translation regulate MAPK-dependent synaptic transmission

**DOI:** 10.1371/journal.pbio.3001969

**Published:** 2023-01-26

**Authors:** Shannon N. Leahy, Chunzhu Song, Dominic J. Vita, Kendal Broadie

**Affiliations:** 1 Department of Biological Sciences, Vanderbilt University and Medical Center, Nashville, Tennessee, United States of America; 2 Department of Cell and Developmental Biology, Vanderbilt University and Medical Center, Nashville, Tennessee, United States of America; 3 Department of Pharmacology, Vanderbilt University and Medical Center, Nashville, Tennessee, United States of America; 4 Vanderbilt Brain Institute, Vanderbilt University and Medical Center, Nashville, Tennessee, United States of America; University of Michigan, UNITED STATES

## Abstract

Noonan syndrome (NS) and NS with multiple lentigines (NSML) cognitive dysfunction are linked to SH2 domain-containing protein tyrosine phosphatase-2 (SHP2) gain-of-function (GoF) and loss-of-function (LoF), respectively. In *Drosophila* disease models, we find both SHP2 mutations from human patients and *corkscrew* (*csw*) homolog LoF/GoF elevate glutamatergic transmission. Cell-targeted RNAi and neurotransmitter release analyses reveal a presynaptic requirement. Consistently, all mutants exhibit reduced synaptic depression during high-frequency stimulation. Both LoF and GoF mutants also show impaired synaptic plasticity, including reduced facilitation, augmentation, and post-tetanic potentiation. NS/NSML diseases are characterized by elevated MAPK/ERK signaling, and drugs suppressing this signaling restore normal neurotransmission in mutants. Fragile X syndrome (FXS) is likewise characterized by elevated MAPK/ERK signaling. Fragile X Mental Retardation Protein (FMRP) binds *csw* mRNA and neuronal Csw protein is elevated in *Drosophila fragile X mental retardation 1* (*dfmr1*) nulls. Moreover, phosphorylated ERK (pERK) is increased in *dfmr1* and *csw* null presynaptic boutons. We find presynaptic pERK activation in response to stimulation is reduced in *dfmr1* and *csw* nulls. *Trans*-heterozygous *csw*/+; *dfmr1*/+ recapitulate elevated presynaptic pERK activation and function, showing FMRP and Csw/SHP2 act within the same signaling pathway. Thus, a FMRP and SHP2 MAPK/ERK regulative mechanism controls basal and activity-dependent neurotransmission strength.

## Abstract 2

This study shows that the RNA-binding protein FMRP binds the Csw/SHP2 transcript, which in turn regulates presynaptic MAPK/ERK signaling and neurotransmission. This mechanistically links previously two previously unconnected cognitive disorders, Noonan syndrome and Fragile X syndrome.

## Introduction

Noonan syndrome (NS) is an autosomal dominant genetic disorder caused by mutations in the mitogen-activated protein kinase (MAPK) pathway [1,2]. Missense mutations within the *protein tyrosine phosphatase non-receptor type 11* (*PTPN11*) gene account for >50% of all disease cases [3]. In both patients and disease models, the MAPK pathway is hyperactivated by NS gain-of-function (GoF) mutations that disrupt the auto-inhibition mechanism between the catalytic protein tyrosine phosphatase domain and N-Src homology-2 (SH2) domain of the *PTPN11* encoded SH2 domain-containing protein tyrosine phosphatase-2 (SHP2; [4,5]). In the NS with multiple lentigines (NSML) disease state, *PTPN11* loss-of-function (LoF) mutations decrease protein tyrosine phosphatase domain catalytic activity, but the mutants nevertheless maintain a more persistently active enzyme state with temporally inappropriate SHP2 function, causing elevated MAPK pathway hyperactivation similar to the GoF disease condition [6]. Consequently, NS and NSML patients share a great many symptoms associated with elevated MAPK signaling, including cognitive dysfunction (approximately 30% of cases) as well as long-term memory (LTM) impairments [7,8]. The *Drosophila* NS (GoF) and NSML (LoF) disease models from mutation of the *corkscrew* (*csw*) homolog likewise both increase MAPK activation, with GoF and LoF also phenocopying each other [9,10]. *Drosophila* LTM training generates repetitive waves of *csw*-dependent neural MAPK activation, with the LTM spacing effect misregulated by *csw* manipulations [11]. *PTPN11* GoF and LoF mutations from human patients transgenically introduced into the *Drosophila* model provide a powerful new means to compare with *csw* GoF and LoF mutants in the dissection of conserved neuronal requirements [12].

Fragile X syndrome (FXS) is similarly well characterized by hyperactivated MAPK signaling within neurons [13], and the causal Fragile X Mental Retardation Protein (FMRP) RNA-binding translational regulator is proposed to directly bind *PTPN11/SHP2* mRNA [14,15]. FMRP also binds many other neuronal transcripts [16] and could interact with SHP2 in multiple ways to coregulate the MAPK pathway. Moreover, like the NS and NSML disease states, FXS is likewise a cognitive disorder and the leading heritable cause of intellectual disability [16]. Like NS and NSML, the *Drosophila* FXS disease model also manifests strongly impaired LTM consolidation [17,18]. Mechanistically, MAPK signaling is well known to modulate glutamatergic synaptic neurotransmission strength via the control of presynaptic vesicle trafficking dynamics and glutamate neurotransmitter release probability [19]. Consistently, FMRP is also well characterized to regulate glutamatergic synaptic neurotransmission, including presynaptic release properties and activity-dependent functional plasticity [20]. Importantly, treatment with the MAPK inhibitor Lovastatin corrects hippocampal hyperexcitability in the mouse FXS disease model and ameliorates behavioral symptoms in human FXS patients [21,22]. In the *Drosophila* FXS disease model, *dfmr1* null mutants show elevated presynaptic glutamate release underlying increased neurotransmission strength [17], as well as activity-dependent hyperexcitability and cyclic increases in glutamate release during sustained high-frequency stimulation trains [23]. Based on this broad foundation, we hypothesized that FMRP regulates PTPN11 (SHP2)/Csw translation to modulate presynaptic MAPK signaling, which, in turn, controls presynaptic glutamate release probability to determine both basal neurotransmission strength and activity-dependent synaptic plasticity.

To investigate this hypothesis, we utilized the *Drosophila* neuromuscular junction (NMJ) glutamatergic model synapse with the combined use of NS, NSML, and FXS disease models. We first tested both LoF and GoF conditions in both (1) *csw* mutants and (2) transgenic human *PTPN11* lines. In two-electrode voltage-clamp (TEVC) electrophysiological recordings, all of these mutant conditions elevate synaptic transmission. We next employed cell-targeted RNAi and spontaneous miniature excitatory junction current (mEJC) recordings to find Csw/SHP2 specifically inhibits presynaptic glutamate release probability. We next tested activity-dependent synaptic transmission using high-frequency stimulation (HFS) depression assays to show that the mutants display heightened transmission resiliency, consistent with elevated presynaptic function. We discovered that both LoF and GoF mutations impair presynaptic plasticity, with decreased short-term facilitation, maintained augmentation and post-tetanic potentiation (PTP), supporting altered presynaptic function. Consistent with elevated MAPK signaling in NS, NSML, and FXS disease models, feeding with MAPK-inhibiting drugs (Trametinib and Vorinostat) corrects synaptic transmission strength in mutants. As predicted, we found that FMRP binds *csw* mRNA and that FMRP loss increases Csw protein levels. Both *dfmr1* and *csw* nulls display elevated phosphorylated ERK (pERK) in presynaptic boutons. Importantly, *trans*-heterozygous double mutants (*csw*/+; *dfmr1*/+) exhibit presynaptic MAPK signaling and neurotransmitter release phenotypes, indicating FMRP and Csw/SHP2 operate to control MAPK/ERK signaling and synaptic function. These discoveries link previously unconnected disease states NS, NSML, and FXS via a presynaptic MAPK/ERK regulative mechanism controlling glutamatergic transmission.

## Results

### Corkscrew/PTPN11 loss and gain of function mutations both increase synaptic transmission

**Fig 1:**
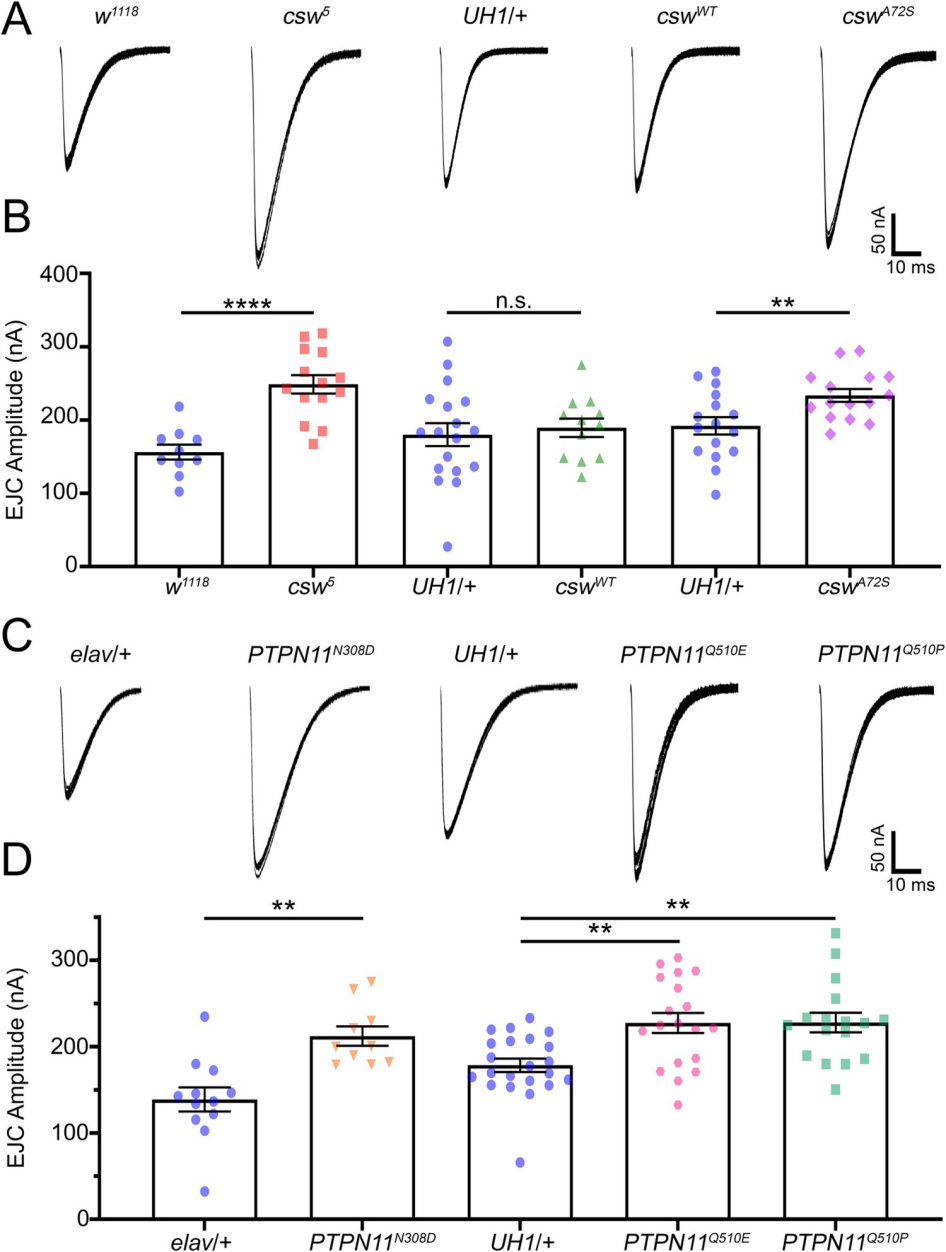
Both loss- and gain-of-function *csw*/*PTPN11* mutants elevate NMJ transmission. TEVC recordings of nerve-stimulated evoked neurotransmission in both LoF and GoF mutations of *Drosophila csw* and human *PTPN11* mutations from NS/NSML patients. (**A)** Representative EJC traces for the *csw* mutant comparisons showing 10 superimposed evoked synaptic responses (1.0 mM Ca^2+^) from *w*^*1118*^ genetic background control, *csw*^*5*^ null mutant, transgenic driver control (*UH1*-Gal4/*w*^*1118*^), wild-type *csw* (*UH1*-Gal4>*csw*^*WT*^), and *csw*^*A72S*^ GoF mutant (*UH1*-Gal4>*csw*^*A72S*^). (**B)** Quantification of the mean EJC amplitudes in all 5 genotypes using two-sided *t* tests. (**C)** Representative evoked EJC traces for the human patient *PTPN11* mutations showing 10 superimposed responses in paired control (*elav*-Gal4/*w*^*1118*^) and GoF mutant (*elav*-Gal4>*PTPN11*^*N308D*^; left), and control (*UH1*-Gal4/*w*^*1118*^) and LoF mutants (*UH1-Gal4*>*PTPN11*^*Q510E*^ and PTPN11^Q510P^; right). (**D)** Quantification of the mean EJC amplitudes in all 5 genotypes using two-sided *t* test, Kruskal–Wallis and Dunn’s multiple comparisons. The scatter plots show all of the individual data points as well as mean ± SEM. *N =* number of NMJs. Significance shown as: *p* > 0.05 (not significant, n.s.), *p* < 0.001 (**) and *p* < 0.0001 (****). The data underlying this figure can be found in S1 Data. *csw*, *corkscrew*; EJC, excitatory junction current; GoF, gain-of-function; LoF, loss-of-function; NMJ, neuromuscular junction; NS, Noonan syndrome; NSML, NS with multiple lentigines; *PTPN11*, protein tyrosine phosphatase non-receptor type 11; TEVC, two-electrode voltage-clamp.

NS and NSML patients often exhibit cognitive deficits [3], which we hypothesized may arise from altered synaptic transmission. To systematically test this hypothesis, we assay both *Drosophila* NS/NSML disease models of *csw* LoF and GoF [9,10,24], as well as *PTPN11* mutations from human patients, including both LoF and GoF point mutants [12]. First, we use *csw*^*5*^, a protein null LoF mutant [24], together with UAS-*csw*^*WT*^ for wild-type Csw overexpression [25] and UAS-*csw*^*A72S*^ as a constitutive GoF mutation [9,11]. Second, we use human patient mutations *PTPN11*^*N308D*^, *PTPN11*^*Q510E*^, and *PTPN11*^*Q510P*^ to capture the range of NS/NSML disease heterogeneity [3,12]. The transgenes were driven with ubiquitous *UH1*-Gal4 or neuronal *elav*-Gal4. The NMJ glutamatergic synapse is used to assay disease model neurotransmission in all variants [26,27]. Employing TEVC recording, we compare mutants to genetic background control (*w*^*1118*^) and transgenic lines to driver controls (*UH1*-Gal4/*w*^*1118*^ and *elav*-Gal4/*w*^*1118*^). We test excitatory junction current (EJC) responses driven by motor nerve suction electrode stimulation (0.5 ms suprathreshold stimuli, 0.2 Hz) onto the voltage-clamped (−60 mV) ventral longitudinal muscle 6 in abdominal segments 3/4 [28]. Each data point is the average of 10 sequentially evoked EJC responses recorded in 1 mM [Ca^2+^] from the same NMJ terminal. Representative recordings and quantified results for all of these comparisons are shown in Fig 1.

In genetic background controls (*w*^*1118*^), nerve stimulation causes consistent, high-fidelity neurotransmission (Fig 1A, left). In comparison, *csw*^*5*^ LoF mutants display highly elevated synaptic function with an obvious increase in amplitude (Fig 1A, second from left). Quantified measurements show *csw*^*5*^ EJC amplitudes (248.80 ± 12.51 nA, *n =* 14) strongly elevated compared to controls (156.30 ± 10.28 nA, *n* = 10), which is a significant increase (*p* < 0.0001, two-sided *t* test; Fig 1B). Since NSML (LoF) and NS (GoF) disease states manifest closely parallel phenotypes, we next examined transgenically driven wild-type *csw* (*csw*^*WT*^) and the GoF mutant (*csw*^*A72S*^). In transgenic ubiquitous driver controls (*UH1*-Gal4/*w*^*1118*^), nerve stimulation drives transmission comparable to the genetic background alone (Fig 1A, middle). Likewise, *csw*^*WT*^ overexpression results in no detectable alteration in synaptic strength, with amplitudes comparable to controls (Fig 1A, second from right). In sharp contrast, the GoF mutant *csw*^*A72S*^ exhibits a consistent elevation in transmission amplitude (Fig 1A, right). Quantification shows the *UH1*-Gal4/*w*^*1118*^ control amplitude (180.10 ± 15.74 nA, *n =* 18) is comparable to *UH1*-Gal4>*csw*^*WT*^ (189.50 ± 12.52 nA, *n* = 12), with no significant difference in transmission (*p* = 0.671, two-sided *t* test; Fig 1B, middle). The *csw*^*A72S*^ GoF mutation causes significantly elevated neurotransmission. Quantified measurements show *csw*^*A72S*^ EJC amplitudes (233.70 ± 8.71 nA, *n* = 15) are strongly increased compared to *UH1*-Gal4/*w*^*1118*^ driver controls (192.10 ± 11.86 nA, *n* = 16), a significant elevation (*p* = 0.009, two-sided *t* test; Fig 1B). This increased neurotransmission is independent of changes in NMJ architecture (S1A Fig), including muscle size (S1B Fig), NMJ area (S1C Fig), branching (S1D Fig), and bouton number (S1E Fig), which show no significant changes. The elevated neurotransmission is also independent of changes in synapse number (S2A Fig), including active zone density (S2B Fig), postsynaptic glutamate receptors (S2C Fig), and synaptic apposition (S2D Fig), which are similarly unaltered. Expressing *csw*^*WT*^ in the *csw*^*5*^ null restores neurotransmission to the control levels (S3A and S3B Fig), indicating phenotype specificity. We therefore conclude that *csw* LoF and GoF increase glutamatergic synaptic transmission, comparable to the phenocopy of NS/NSML disease state symptoms in human patients.

To further test effects, we next assayed *PTPN11* patient mutations. Compared to transgenic controls, all the *PTPN11* mutations cause clearly strengthened synaptic function (Fig 1C). The NS *PTPN11*^*N308D*^, NSML *PTPN11*^*Q510E*^, and NSML *PTPN11*^*Q510P*^ mutations all display consistent EJC elevations compared to the controls, similar to LoF/GoF *csw* animals (compare Fig 1A and 1C). For the GoF condition, the human *PTPN11*^*N308D*^ mutation is driven only in neurons (*elav*-Gal4) since ubiquitous expression results in lethality complications. Quantification compared to neuronal driver control (*elav*-Gal4/w^1118^) EJC amplitude (138.70 ± 13.95 nA, *n =* 12) shows NS (GoF) *PTPN11*^*N308D*^ EJC amplitude (212.20 ± 11.13 nA, *n* = 10) is significantly elevated (*p* = 0.001, two-sided *t* test, Fig 1D, left). The patient-derived *PTPN11* LoF mutations similarly display increased transmission amplitudes, including *PTPN11*^*Q510E*^ (227.40 ± 11.64 nA, *n* = 19) and *PTPN11*^*Q510P*^ (227.90 ± 11.28 nA, *n* = 17) compared to the matched ubiquitous driver controls (*UH1*-Gal4/*w*^*1118*^; 178.40 ± 7.73 nA, *n* = 22). These changes are significant both together (*p* = 0.0006, Kruskal–Wallis; Fig 1D, right) and when compared individually for both *PTPN11*^*Q510E*^ (*p* = 0.004, Dunn’s multiple comparison; Fig 1D) and *PTPN11*^*Q510P*^ (*p* = 0.003, Dunn’s multiple comparisons; Fig 1D). The patient *PTPN11* mutants are not different from each other (*p* > 0.999, Dunn’s multiple comparisons; Fig 1D). Additionally, *PTPN11*^*WT*^ overexpression results in no detectable alteration in synaptic strength, with amplitudes comparable to controls (S3C and S3D Fig). Taken together, these findings indicate that both *Drosophila csw* and human homolog *PTPN11* significantly limit neurotransmission strength. EJCs are elevated with both LoF and GoF, but not by simple overexpression. The next pressing question was to determine whether synaptic strengthening is due to increased presynaptic glutamate release, postsynaptic glutamate receptor responsiveness, or both together.

### Corkscrew/PTPN11 controls presynaptic transmission by altering glutamate release probability

**Fig 2:**
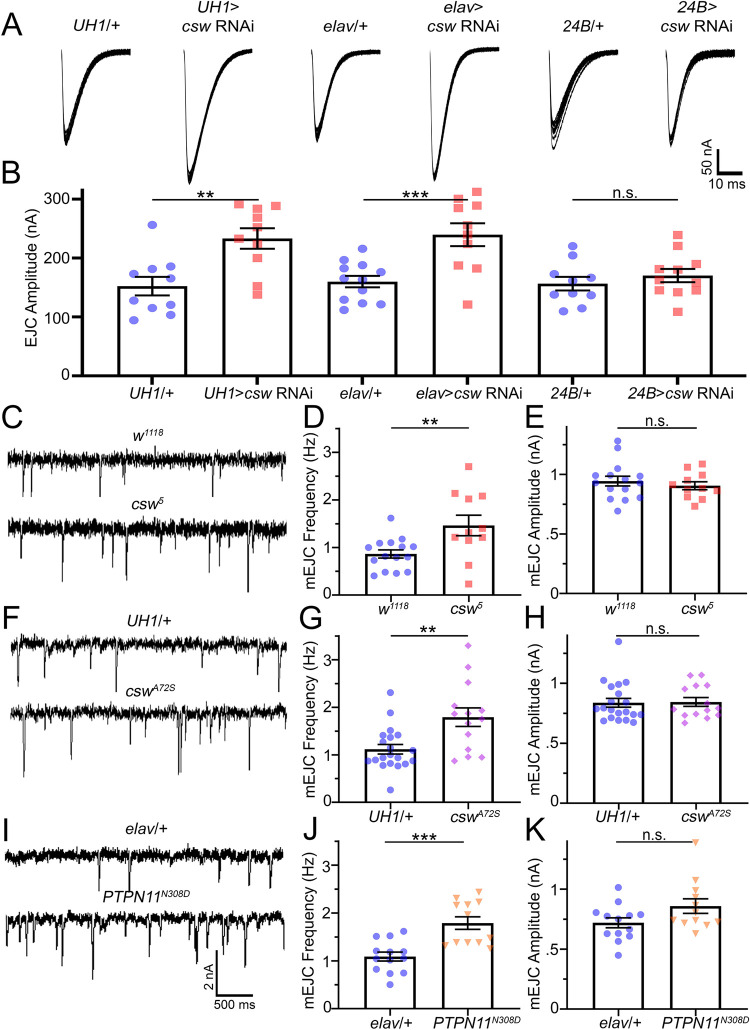
Targeted neuronal *csw* knockdown increases presynaptic neurotransmission. Nerve stimulation–evoked recordings based on *csw* RNAi expressed ubiquitously (*UH1*-Gal4) or targeted to neurons (*elav*-Gal4), or muscles (*24B*-Gal4). (**A)** Representative EJC traces showing 10 superimposed responses (1.0 mM Ca^+2^) from control (*UH1*-Gal4/TRiP) vs. *csw* RNAi; control (*elav*-Gal4/TRiP) vs. *csw* RNAi; and control (*24B*-Gal4/TRiP) vs. *csw* RNAi. (**B)** Quantification of EJC amplitudes using two-sided *t* tests. (**C)** Representative mEJC traces (1.0 mM Ca^2+^) in genetic background control (*w*^*1118*^, top) and *csw*^*5*^ null (bottom). (**D)** Quantification of the mEJC frequencies using a two-sided *t* test. (**E)** Quantification of the mEJC amplitudes using a two-sided *t* test. (**F)** Sample mEJC recordings from the driver control (*UH1*-Gal4/*w*^*1118*^; top) compared to *csw*^*A72S*^ GoF *(UH1*-Gal4*>csw*^*A72S*^; bottom). **(G)** Quantification of the mEJC frequencies using a two-sided *t* test. (**H)** Quantification of mEJC amplitudes using Mann–Whitney test. (**I)** Sample mEJC recordings in control (*elav*-Gal4/*w*^*1118*^; top) compared to *PTPN11*^*N308D*^ GoF (*elav*-Gal4*>PTPN11*^*N308D*^; bottom). **(J)** Quantification of the mEJC frequency using a Mann–Whitney test. (**K)** Quantification of mEJC amplitude using a Mann–Whitney test. Scatter plots show all the data points and mean ± SEM. *N =* number of NMJs. Significance: *p* > 0.05 (not significant, n.s.), *p* < 0.05 (*), *p* < 0.001 (**), and *p* < 0.0001 (***). The data underlying this figure can be found in S1 Data. *csw*, corkscrew; EJC, excitatory junction current; mEJC, miniature EJC; NMJ, neuromuscular junction; RNAi, RNA interference.

Our next objective was to determine where Corkscrew acts to mediate synaptic changes in neurotransmission strength. To test requirements, we knocked down *csw* expression through RNA interference (RNAi) driven in the different cells contributing to the NMJ, including the presynaptic motor neuron and postsynaptic muscle [29]. We used targeted transgenic RNAi against *csw* (BDSC 33619; [30]) to test each cell-specific function. This line is from the Harvard Transgenic RNAi Project (TRiP), which provides a background control stock (BDSC 36303) containing all components except the UAS-RNAi [31]. To test RNAi efficacy and replication of *csw*^*5*^ null phenotypes, we first used the ubiquitous *daughterless* UH1-Gal4 driver. To separate cellular requirements, we used neuronal *elav*-Gal4 and muscle *24B*-Gal4-specific drivers, each compared to their respective driver alone transgenic controls. With each RNAi knockdown, we once again utilized TEVC recordings of evoked EJC neurotransmission to measure synaptic strength. To further test *csw* functional roles, we analyzed spontaneous release events by assessing changes in both frequency and amplitude with miniature EJC (mEJC) recordings [28]. Changes in the mEJC frequency are correlated with alterations in presynaptic fusion probability, whereas changes in mEJC amplitudes indicate differential postsynaptic glutamate receptor function or altered vesicle size [32,33]. We made continuous mEJC recordings collected over 2 minutes using a gap-free configuration filtered at 10 kHz [28]. Each data point corresponds to the mean mEJC frequency and amplitude of all the recorded release events. Representative recordings and quantified results are shown in Fig 2.

The ubiquitous transgenic driver control (*UH1*-Gal4/TRiP BDSC 36303 control) exhibits neurotransmission indistinguishable from the *w*^*1118*^ genetic background control (Fig 2A, left). Ubiquitous *csw* knockdown (UH1>*csw* RNAi) causes elevated neurotransmission closely consistent with the *csw*^*5*^ null mutant (Fig 2A, second from left), demonstrating RNAi efficacy as well as null phenocopy (compare to Fig 1A, left). The quantified EJC measurements show UH1>*csw* RNAi (233.20 ± 17.45 nA, *n =* 10) to be strongly elevated compared to controls (152.30 ± 15.65 nA, *n* = 10), which is a significant increase (*p* = 0.003, two-sided *t* test; Fig 2B). The neuronal driver control (*elav*-Gal4/TRiP BDSC 36303 control) compared to neuronal-specific knockdown (*elav>csw* RNAi) also shows strong replication of the *csw*^*5*^ null elevated transmission, indicating a primary *csw* requirement in the presynaptic neuron (Fig 2A, middle pair). Quantified measurements show *elav>csw* RNAi EJC amplitude (239.70 ± 19.45 nA, *n =* 10) also strongly increased compared with the *elav*-Gal4/TRiP driver controls (159.90 ± 9.68 nA, *n* = 12), which is significant (*p* = 0.001, two-sided *t* test; Fig 2B, middle). In contrast, targeted muscle RNAi knockdown (*24B*>*csw* RNAi) does not cause any change in evoked neurotransmission compared to the muscle driver control alone (*24B*-Gal4/TRiP BDSC 36303; Fig 2A, right pair), signifying that postsynaptic Csw does not detectably change synaptic function. When quantified, *24B*-Gal4/TRiP (156.50 ± 11.41 nA, *n =* 10) is comparable to *24B*>*csw* RNAi (170.30 ± 11.24 nA, *n =* 11), with no significant change in amplitude (*p* = 0.401, two-sided *t* test; Fig 2B, right). These findings indicate a primary *csw* requirement in presynaptic neurons regulating glutamate neurotransmitter release.

To further test pre- versus postsynaptic requirements, we next analyzed spontaneous mEJC release events. Compared to genetic background controls (*w*^*1118*^), *csw*^*5*^ null mutants exhibit an obvious increase in mEJC frequency, without any detectable alteration in amplitudes (Fig 2C). When quantified, mEJC frequency in *csw*^*5*^ nulls (1.46 ± 0.22 Hz, *n =* 11) is increased compared to controls (0.86 ± 0.086 Hz, *n* = 15), a significant elevation (*p* = 0.009, two-sided *t* test; Fig 2D). There is no significant change in mEJC amplitudes (*p* = 0.489, two-sided *t* test; Fig 2E). Like the null mutant, GoF *csw*^*A72S*^ animals show increased mEJC frequency compared to controls, with no increase in amplitude (Fig 2F). When quantified, UH1>*csw*^*A72S*^ (1.79 ± 0.19 Hz, *n* = 14) have increased mEJC frequency compared to controls (1.12 ± 0.10 Hz, *n* = 20), which is a significant elevation (*p* = 0.002, two-sided *t* test; Fig 2G). Quantification shows no significant change in mEJC amplitudes (*p* = 0.796, Mann–Whitney; Fig 2H). Similarly, patient-derived *PTPN11*^*N308D*^ mutants display increased mEJC frequency with no change in amplitude (Fig 2I). Quantification shows *PTPN11*^*N308D*^ frequency (1.79 ± 0.13 Hz, *n* = 12) increased versus controls (1.09 ± 0.09 Hz, *n* = 13), which is a significant elevation (*p* = 0.001, Mann–Whitney; Fig 2J). There is no significant change in amplitudes (*p* = 0.168, Mann–Whitney; Fig 2K). These findings indicate that both LoF and GoF mutations alter neurotransmission by increasing presynaptic glutamate release rate. We confirmed results further by testing mEJCs in different RNAi conditions. We find mEJC frequencies increased with ubiquitous *csw* RNAi (S4A and S4B Fig) and neuron-targeted *csw* RNAi (S4D and S4E Fig), but no change with muscle-specific RNAi (S4G and S4H Fig). None of these manipulations alter mEJC amplitude (S4C, S4F and S4I Fig). Taken together with targeted RNAi results, we conclude that a neuronal requirement regulates glutamate release from the presynaptic terminal. Quantal content determined by dividing EJC amplitude by mean mEJC amplitude shows elevated quantal content in the mutants (S5A Fig) as well as ubiquitous/neuronal *csw* RNAi (S5B Fig). Moreover, *PTPN11* LoF patient mutations driven neuronally phenocopy all GoF defects, including elevated neurotransmission (S6A and S6B Fig) and increased presynaptic fusion (S6C and S6D Fig), but no change in mEJC amplitude (S6E Fig), consistent with the increase in quantal content (S6F Fig). This suggested that stimulation paradigms challenging neurotransmission maintenance should reveal changes in vesicle release dynamics in the absence of *csw/PTPN11* function.

### Corkscrew/PTPN11 regulates high-frequency stimulation synaptic depression

**Fig 3:**
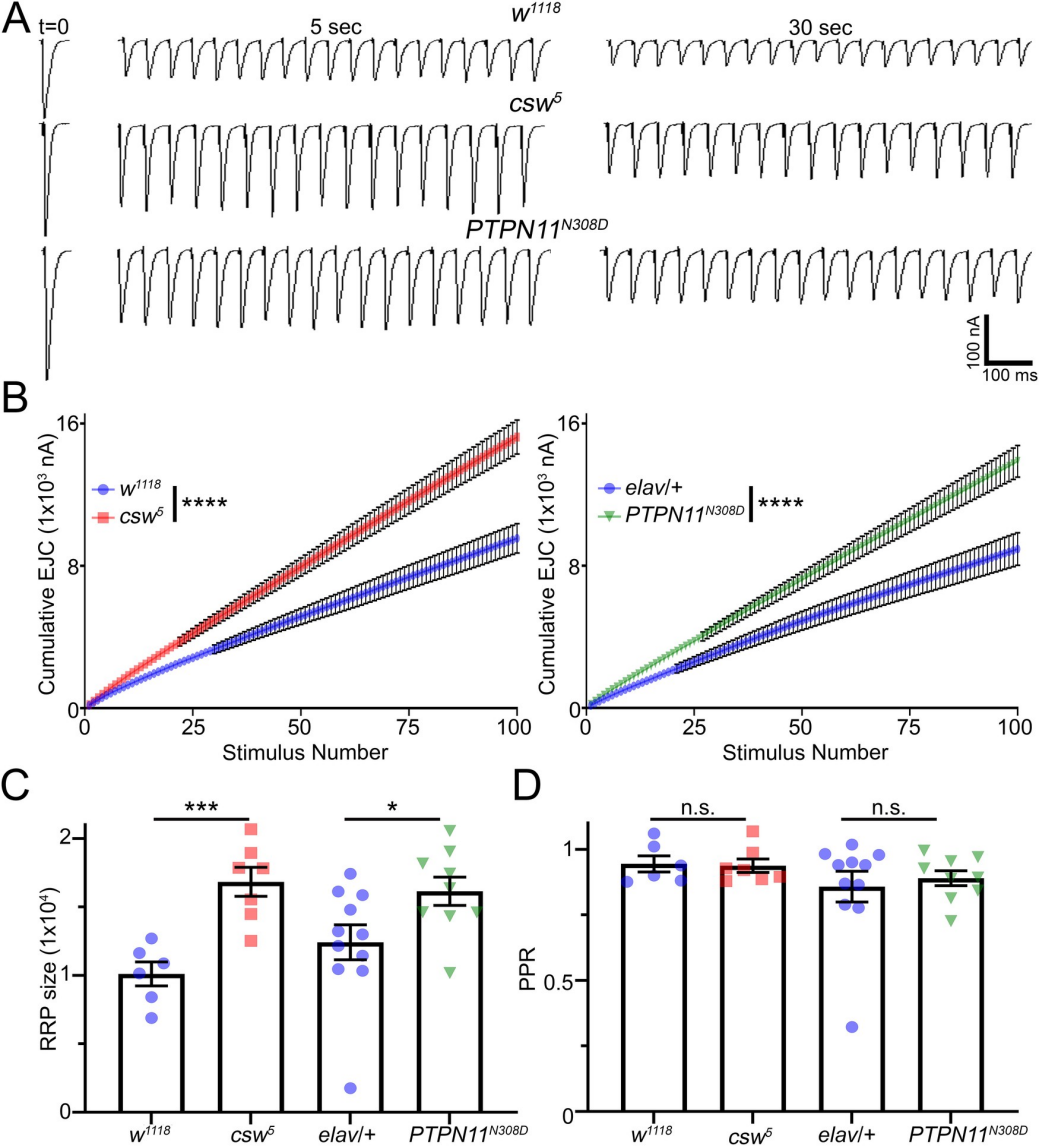
HFS transmission depression ameliorated in *csw* nulls. Prolonged HFS drives progressive synaptic amplitude depression over several minutes of continuous recording at 20 Hz (1mM Ca^+2^). (**A)** Representative nerve-stimulated EJC traces at the basal frequency (t = 0) and indicated time points during the HFS train for genetic background control (*w*^*1118*^, top), *csw* null (*csw*^*5*^, middle), and *PTPN11*^*N308D*^ GoF mutant (*elav*-Gal4*>PTPN11*^*N308D*^; bottom). (**B)** Quantification of cumulative EJC amplitudes over the first 100 stimulations via nonlinear regression exponential for each pair tested using extra sum-of-squares F tests. (**C)** Quantification of the RRP of *w*^*1118*^ and *csw*^*5*^ (two-sided *t* test) and *elav*-Gal4/*w*^*1118*^ and *PTPN11*^*N308D*^ (Mann–Whitney). (**D)** Quantification of the PPR of *w*^*1118*^ and *csw*^*5*^ (two-sided *t* test) and *elav*-Gal4/*w*^*1118*^ and *PTPN11*^*N308D*^ (Mann–Whitney). Scatter plots show all data points and mean ± SEM. *N =* number of NMJs. Significance: *p* < 0.05 (*), *p* < 0.001 (**), *p* < 0.001 (***), and *p* < 0.0001 (****). The data underlying this figure can be found in S1 Data. *csw*, corkscrew; EJC, excitatory junction current; HFS, high-frequency stimulation; NMJ, neuromuscular junction; PPR, paired-pulse ratio; RRP, readily releasable pool.

To further investigate how *csw/PTPN11* affects presynaptic neurotransmission strength, we stimulated at a heightened frequency that has been shown to cause synaptic depression over a time course of several minutes [34–36]. Synaptic depression occurs when HFS causes synaptic vesicles to be released at a faster rate than they can be replenished in presynaptic boutons [34,37]. Based on published HFS protocols for the *Drosophila* NMJ [34,36,38], we compared the genetic background control (*w*^*1118*^), *csw* null LoF mutant (*csw*^*5*^), and patient-derived *PTPN11*^*N308D*^ GoF mutant (*elav*-Gal4*>PTPN11*^*N308D*^) with a HFS paradigm. To determine the baseline EJC amplitudes, we first stimulated for 1 minute under basal conditions (0.5 ms suprathreshold stimuli at 0.2 Hz in 1.0 mM external [Ca^2+^]). We then stimulated at 100X greater frequency (20 Hz) for 5 minutes while continuously recording EJC responses. This sustained HFS train causes progressively decreased neurotransmission over time (depression). HFS transmission was quantified to analyze the synaptic vesicle readily releasable pool (RRP) and paired-pulse ratio (PPR) release probability. Representative HFS recordings and quantified results are shown in Figs 3 and S7.

During HFS, *w*^*1118*^ controls exhibit a steady decrease in EJC amplitudes throughout the train (Fig 3A, top). The *PTPN11*^*N308D*^ GoF mutants and *csw*^*5*^ LoF nulls show stronger maintained EJC amplitudes over time and prolonged resistance to depression (Figs 3A and S7A). RRP size was calculated by dividing the cumulative EJCs during the first 100 responses by mean mEJC amplitudes [39]. There is a sustained elevated response in both LoF and GoF mutants (Fig 3B). When compared with nonlinear regression and extra sum-of-squares, the stimulation train profiles are significantly greater for both LoF (*p* < 0.0001, F_(2,1296)_ = 1064) and GoF (*p* < 0.0001, F_(2,1996)_ = 705.5; Fig 3B) mutants, indicating increased resiliency to depression. The RRP size of *csw*^*5*^ nulls is significantly increased compared to *w*^*1118*^ background controls (*p* = 0.001, two-sided *t* test; Fig 3C, left). Similarly, *PTPN11*^*N308D*^ GoF mutants exhibit an increased RRP compared to transgenic *elav*/+ neuronal driver controls (*p* = 0.047, Mann–Whitney; Fig 3C, right). PPR analyzed for both mutants shows no in change in *csw*^*5*^ nulls (*p* = 0.865, two-sided *t* test; Fig 3D, left) or *PTPN11*^*N308D*^ GoF mutants (*p* = 0.941, Mann–Whitney; Fig 3D, right) compared to their respective controls. The depression resistance continues for 5 minutes of continuous stimulation (S7B Fig). Taken together, these results indicate mutants maintain transmission better with a HFS challenge. We therefore next turned to examining changes in activity-dependent synaptic function under both LoF and GoF mutant conditions.

### Corkscrew/PTPN11 enables short-term plasticity facilitation, augmentation, and potentiation

**Fig 4:**
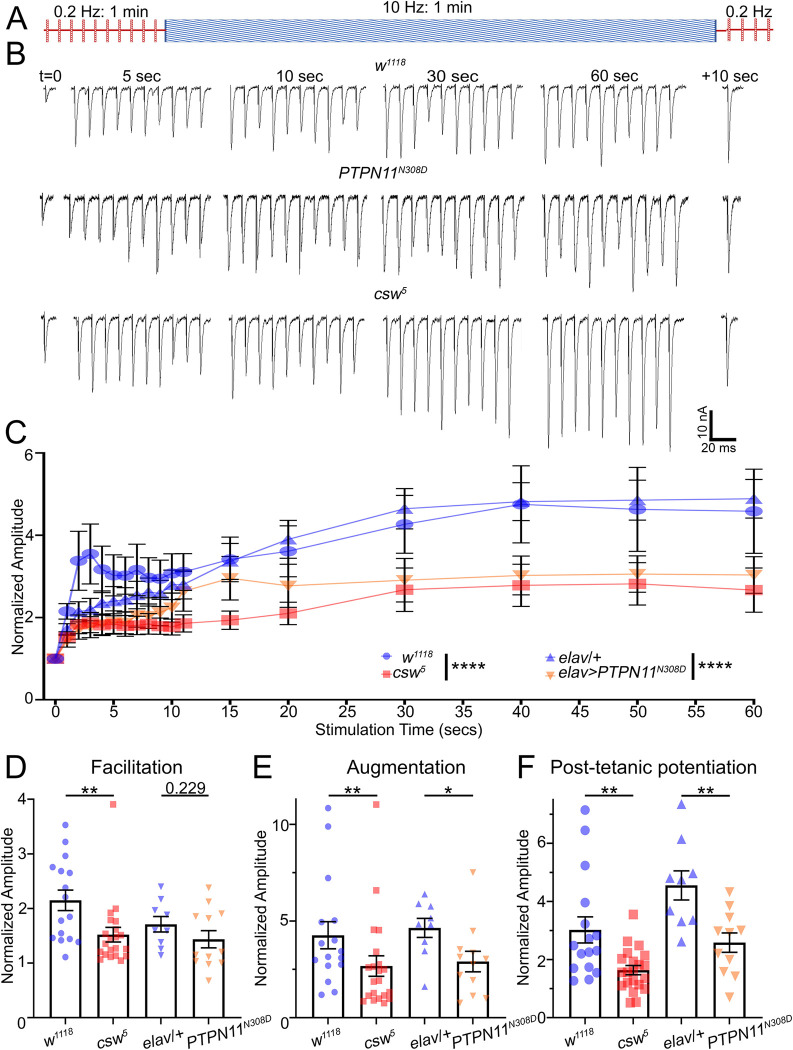
Activity-dependent synaptic plasticity repressed in *csw*/*PTPN11* mutants. Synaptic plasticity during and following a short-term stimulation train to measure facilitation, augmentation, and PTP. (**A)** Stimulation paradigm: 1 minute at 0.2 Hz (0.2 mM Ca^2+^), followed by 1 minute at 10 Hz, and then a return to 0.2 Hz. (**B)** Sample EJC traces at indicated time points during and following the 10 Hz train for control (*w*^*1118*^, top), GoF *PTPN11*^*N308D*^ (*elav*-Gal4*>PTPN11*^*N308D*^; middle), and *csw* null (*csw*^*5*^, bottom). (**C)** Quantification of EJC amplitude during the 10-Hz train normalized to basal EJC amplitude for each genotype. The nonlinear regression exponential for each pair tested using extra sum-of-squares F test. (**D-F)** Quantification of facilitation (1 second, **D**) and augmentation (30 seconds, **E**) during the 10-Hz train, and PTP (10 seconds following train, **F**) normalized to the basal EJC amplitude for each genotype using Mann–Whitney/two-sided *t* tests. Scatter plots show all data points and mean ± SEM. *N =* number of NMJs. Significance: *p* < 0.05 (*), *p* < 0.001 (**), and *p* < 0.0001 (****). The data underlying this figure can be found in S1 Data. *csw*, corkscrew; EJC, excitatory junction current; GoF, gain-of-function; NMJ, neuromuscular junction; PTP, post-tetanic potentiation; *PTPN11*, protein tyrosine phosphatase non-receptor type 11.

Presynaptic activity drives numerous forms of short-term plasticity dependent on release mechanisms [40,41]. In high external [Ca^2+^], strong stimulation results in neurotransmission depression as above, but with reduced external [Ca^2+^], many forms of release strengthening are revealed, including short-term facilitation and maintained augmentation during stimulation trains, and PTP following the train [42–44]. Based on published *Drosophila* plasticity protocols [23], we compared genetic background controls (*w*^*1118*^ or *elav-*Gal4/*w*^*1118*^), *csw* LoF nulls (*csw*^*5*^), and *PTPN11* GoF animals (*elav*-Gal4*>PTPN11*^*N308D*^) with the stimulation paradigm illustrated in Fig 4A. To determine baseline EJC amplitudes, we stimulated at the basal frequency (0.5 ms suprathreshold stimuli/0.2 Hz in 0.2 mM [Ca^2+^]). We then applied a 10-Hz train for 1 minute, before returning to 0.2 Hz for PTP analyses (Fig 4A). In controls, this paradigm drives strong short-term facilitation during the initial stimuli of the train, followed by maintained transmission augmentation for the full duration of the train [42]. Following return to the basal stimulation frequency (0.2 Hz), heightened EJC amplitudes persist during the PTP period (Fig 4B; [42]). We normalized EJC amplitudes during and after the 10-Hz train to the initial mean EJC amplitude to show only transmission changes in response to stimulation. Quantified analyses on *w*^*1118*^ control, *csw*^*5*^ LoF, and *PTPN11*^*N308D*^ GoF mutants were done for facilitation (<1 second), augmentation (>5 seconds), and PTP (following the HFS train). Representative short-term plasticity recordings and quantified results are shown in Fig 4.

Controls exhibit robust synaptic plasticity, including short-term facilitation (<1 second), maintained augmentation (>5 seconds), and persistent PTP (Fig 4C, top two blue lines). With HFS, *w*^*1118*^ controls exhibit a >3-fold amplitude increase in <5 seconds, which strengthens to a 4-fold increase by 30 seconds. After the HFS train, control animals PTP at >2-fold basal transmission. In contrast, this short-term plasticity is strongly repressed in both the *csw*^*5*^ LoF and *PTPN11*^*N308D*^ GoF mutants (Fig 4C, bottom two red lines). When quantified via nonlinear regression and extra sum-of-squares, stimulation train profiles significantly differ for both LoF (*p* < 0.0001, F_(2,662)_ = 38.95) and GoF (*p* < 0.0001, F_(2,374)_ = 25.85; Fig 4C). During initial short-term facilitation (1 second), *w*^*1118*^ controls show much stronger strengthening normalized to basal amplitude (2.15 ± 0.19, *n =* 16) compared to *csw*^*5*^ LoF (1.52 ± 0.14, *n* = 21; *p* = 0.005, Mann–Whitney) and a trending decrease in *PTPN11*^*N308D*^ GoF (1.44 ± 0.16, *n* = 12; *p* = 0.229, two-sided *t* test; Fig 4D). With maintained augmentation during the HFS train (30 seconds), *w*^*1118*^ controls are highly elevated (4.27 ± 0.70, *n* = 16) compared to *csw*^*5*^ LOF (2.67 ± 0.53, *n* = 21; *p* = 0.009, Mann–Whitney) and *PTPN11*^*N308D*^ GOF (2.91 ± 0.53, *n* = 12; *p* = 0.015, Mann–Whitney; Fig 4E). At peak PTP after the HFS train, *w*^*1118*^ controls exhibit a significant increase (3.02 ± 0.45, *n* = 16) compared to *csw*^*5*^ LoF (1.63 ± 0.16, *n* = 21; *p* = 0.003, Mann–Whitney; Fig 4F). Likewise, the *PTPN11*^*N308D*^ GoF (2.58 ± 0.33, *n* = 11) shows significantly decreased PTP compared to *elav-*Gal4/*w*^*1118*^ controls (4.55 ± 0.5, *n* = 9; *p* = 0.003, two-sided *t* test; Fig 4F). These results show a role in presynaptic release dynamics, with altered responses to evoked stimulation. To understand the mechanism of these changes, we next turned to testing the role of MAPK/ERK signaling.

### Elevated Corkscrew/PTPN11 synaptic transmission corrected with pERK inhibitors

**Fig 5:**
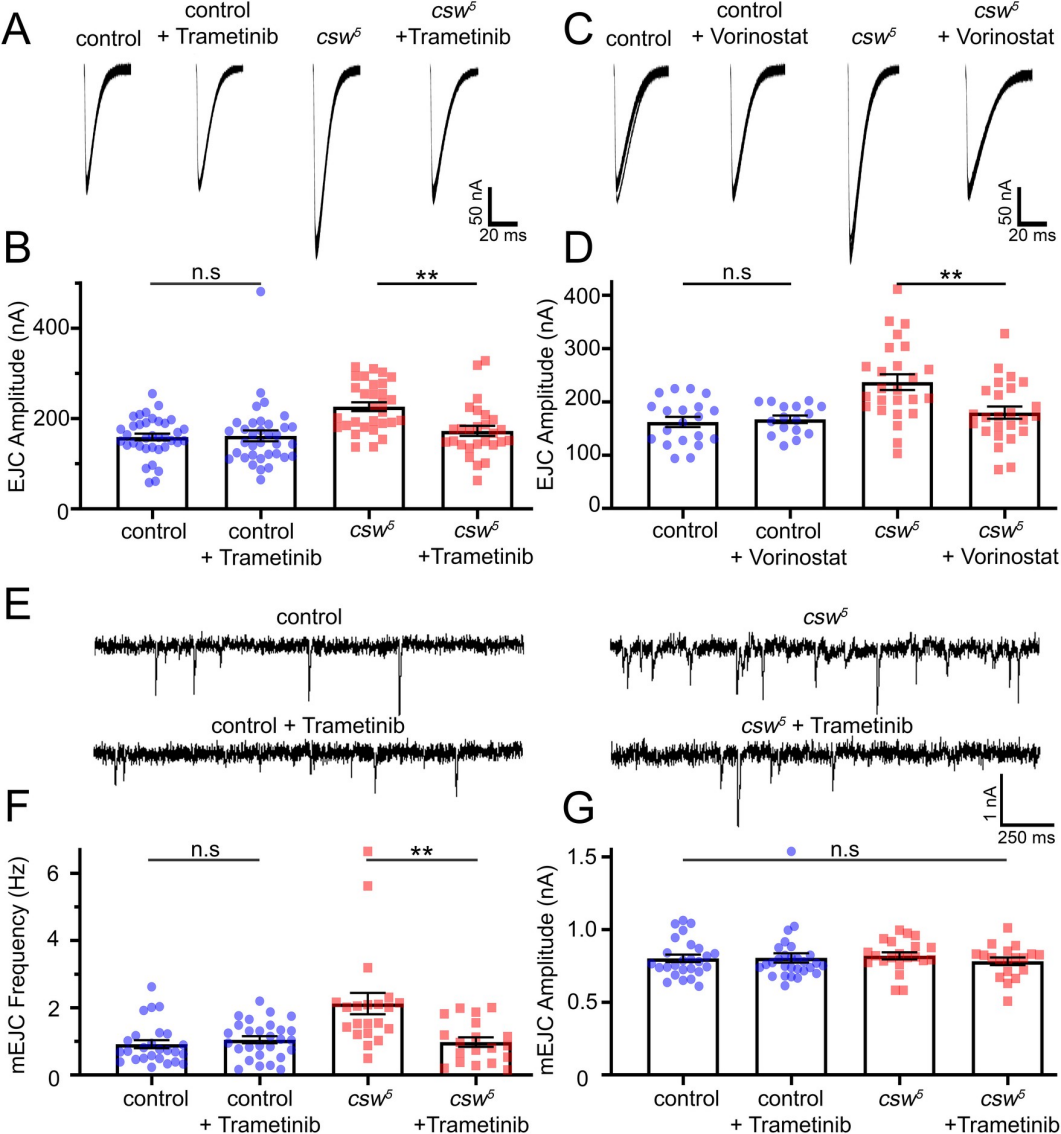
Reducing pERK signaling restores synaptic function in *csw* nulls. TEVC recordings with and without two pERK inhibiting drugs (Trametinib and Vorinostat) comparing the genetic background control (*w*^*1118*^) and *csw* null mutant (*csw*^*5*^). (**A)** Representative EJC traces showing 10 superimposed responses (1.0 mM Ca^+2^) comparing the control (left) and *csw*^*5*^ null mutant (right), with and without Trametinib. (**B)** Quantification of mean EJC amplitudes for all 4 conditions using Kruskal–Wallis followed by Dunn’s multiple comparisons. (**C)** Representative EJC traces comparing the control (left) and *csw*^*5*^ null mutant (right), with and without Vorinostat. (**D)** Quantification of EJC amplitudes for all 4 conditions using one-way ANOVA followed by Tukey’s multiple comparisons. (**E)** Representative mEJC traces (1.0 mM Ca^2+^) in the *w*^*1118*^ control (left) and *csw*^*5*^ null mutant (right), with and without Trametinib. (**F)** Quantification of mEJC frequency in all 4 conditions using a Kruskal–Wallis followed by Dunn’s multiple comparisons. (**G)** Quantification of mEJC amplitudes using a Kruskal–Wallis. Scatter plots show all the data points and the mean ± SEM. *N =* number of NMJs. Significance: *p* > 0.05 (not significant, n.s.) and *p* < 0.001 (**). The data underlying this figure can be found in S1 Data. EJC, excitatory junction current; mEJC, miniature EJC; NMJ, neuromuscular junction; pERK, phosphorylated ERK; TEVC, two-electrode voltage-clamp.

NS and NSML phenotypes are hypothesized to converge due to both LoF/GoF disease states exhibiting constitutively elevated MAPK/ERK signaling [10]. Similarly, we hypothesize the mutant LoF/GoF neurotransmission elevation from heightened glutamate release also occurs downstream of elevated presynaptic MAPK/ERK signaling. To test this hypothesis, we used MAPK/ERK inhibitors (Trametinib and Vorinostat) to assay effects on glutamatergic synaptic function. Trametinib binds and inhibits MEK1/2 [45], resulting in a direct inhibition of MAPK/ERK signaling [12]. Vorinostat acts as a HDAC inhibitor to also inhibit MAPK/ERK signaling [12,46]. Recent work using the *PTPN11* mutations from human patients has highlighted these two drugs as possible treatments for a variety of different NS/NSML mutations [12]. Both drugs are thus interesting not only for their ability to test elevated MAPK/ERK signaling upstream of neurotransmission, but also as possible future treatment avenues. We fed both drugs and then analyzed changes in EJC amplitudes using TEVC recording. For each drug, we compared the background control (*w*^*1118*^) without drug treatments to controls with drug treatments (Trametinib and Vorinostat), as well as the *csw* null mutants (*csw*^*5*^) without drug treatments to nulls with drug treatments (Trametinib and Vorinostat). Quantification of evoked EJC amplitudes in all 8 conditions tests whether each drug changes neurotransmission in control, as well as correction of the null *csw*^*5*^ elevated neurotransmission (Fig 1A). We also analyzed mEJC recordings of the same genotypes to test for correction of *csw*^*5*^ elevated mEJC frequency (Fig 2C and 2D). Representative EJC and mEJC traces and quantified results are shown in Fig 5.

Null *csw*^*5*^ animals fed Trametinib have clearly decreased neurotransmission compared to untreated mutants, with EJC amplitudes comparable to control animals (Fig 5A). Quantification shows untreated controls (159.10 ± 7.35 nA, *n =* 36) and drugged controls (161.70 ± 12.01 nA, *n* = 35) are not significantly different (*p* > 0.99, Dunn’s; Fig 5B). In contrast, c*sw*^*5*^ EJC amplitudes (226.20 ± 9.79 nA, *n* = 30) are significantly increased compared to controls with (*p* < 0.0001, Dunn’s; Fig 5B) and without Trametinib (*p* = 0.001, Dunn’s, Fig 5B). Critically, c*sw*^*5*^ nulls fed Trametinib (172.70 ± 11.37 nA, *n* = 27) are no longer significantly increased from controls with or without Trametinib (*p* > 0.99, Dunn’s) but are significantly decreased compared to the untreated c*sw*^*5*^ nulls (*p* = 0.003, Dunn’s; Fig 5B). Similar results occur with Trametinib treatment of *PTPN11*^*N308D*^ GoF mutants (S8A and S8B Fig). Similarly, Vorinostat fed *csw*^*5*^ nulls have EJC amplitudes restored to the control levels (Fig 5C). Quantification shows controls with (167.20 ± 7.01 nA, *n* = 16) and without (162.30 ± 9.46 nA, *n* = 20) Vorinostat are not significantly different (*p* = 0.994, Tukey’s; Fig 5D). In contrast, *csw*^*5*^ mutants (237.0 ± 14.72 nA, *n* = 25) are significantly increased versus controls with (*p* = 0.001, Tukey’s) and without (*p* = 0.0001, Tukey’s) Vorinostat (Fig 5D). Null *csw*^*5*^ fed Vorinostat (179.70 ± 11.55 nA, *n* = 25) are not significantly elevated compared to controls with (*p* = 0.897) and without (*p* = 0.727) Vorinostat but are significantly decreased compared to untreated *csw*^*5*^ nulls (*p* = 0.003, Tukey’s; Fig 5D). Trametinib decreases mEJC frequency in *csw*^*5*^ nulls compared to untreated mutants, to levels matching controls (Fig 5E). Quantification shows untreated (0.92 ± 0.12 Hz *n* = 26) and drugged (1.05 ± 0.103 Hz, *n* = 28) controls are not significantly different (*p* > 0.99, Dunn’s; Fig 5F). In contrast, c*sw*^*5*^ mEJC frequency (2.13 ± 0.32 Hz, *n* = 21) is significantly increased compared to controls (no drug, *p* < 0.0001, Dunn’s; Trametinib, *p* = 0.003, Dunn’s; Fig 5F). Critically, c*sw*^*5*^ nulls fed Trametinib (0.98 ± 0.14 Hz, *n* = 19) are no longer significantly increased from controls with or without the drug (*p* > 0.99, Dunn’s) but are significantly decreased compared to untreated c*sw*^*5*^ mutants (*p* = 0.001, Dunn’s; Fig 5F). There are no changes in mEJC amplitude (*p* = 0.437, Kruskal–Wallis; Fig 5G). Thus, decreasing MAPK/ERK signaling restores presynaptic neurotransmission in *csw*^*5*^ animals. We therefore next aimed to identify the upstream mechanism controlling this regulation.

### FMRP binds *csw* mRNA to suppress Csw protein expression upstream of MAPK/ERK signaling

**Fig 6:**
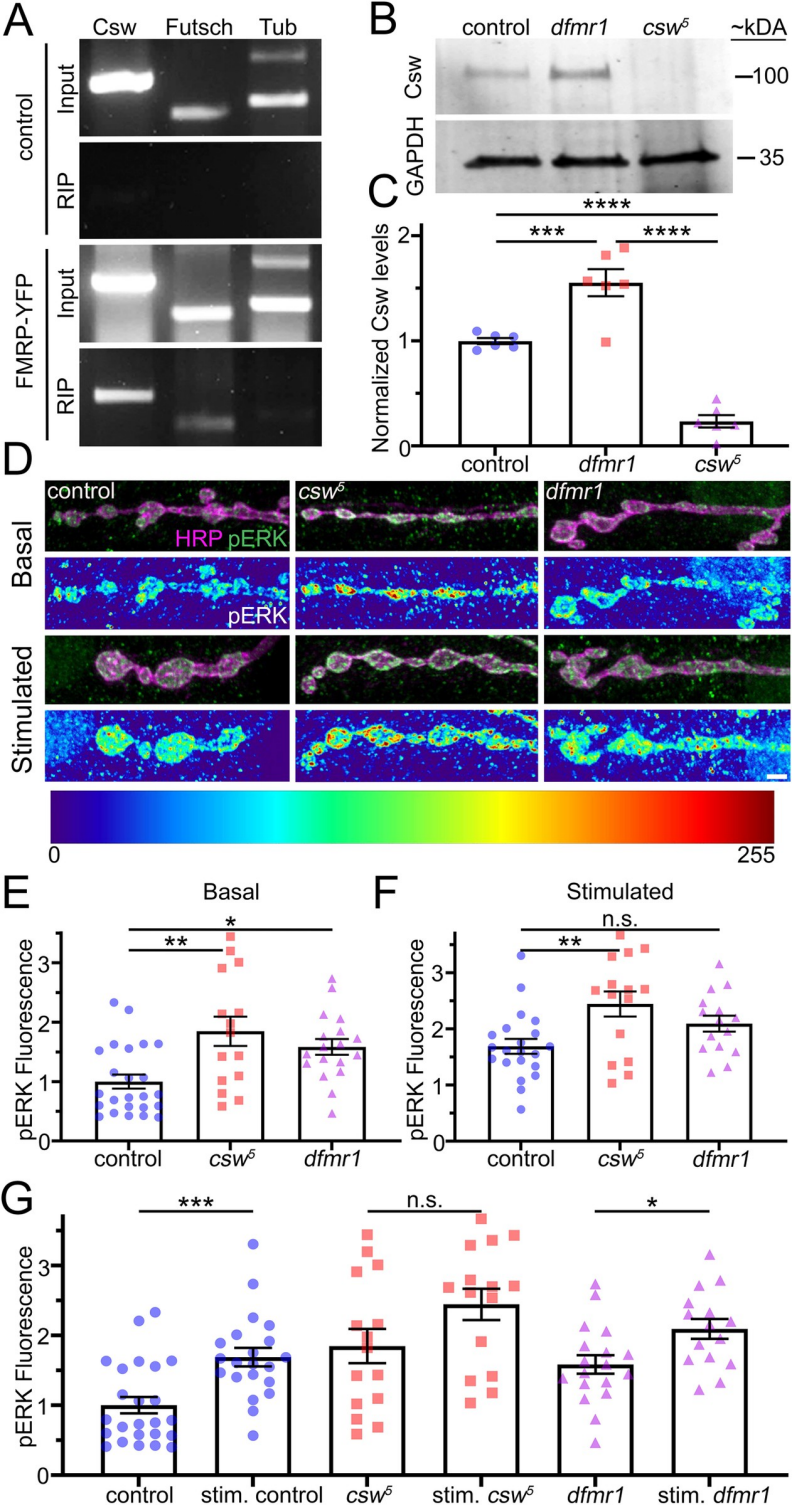
FMRP binds *csw* mRNA to elevate neuronal Csw and presynaptic pERK levels. **(A)** RIP control (Tubby::GFP, top) and FMRP (FMRP::YFP, bottom), with *csw*, *futsch* (positive control), and α*-tubulin* (negative control) RNAs. (**B)** Western blot for Csw (100 kDa, top) and GAPDH control (35 kDA, bottom) *w*^*1118*^ control, *dfmr1*^*50M*^ null, and *csw*^*5*^ null. (**C)** Quantification of Csw levels normalized to GAPDH using one-way ANOVA followed by Tukey’s multiple comparisons. **(D)** Representative NMJ images of *w*^*1118*^ control, *csw*^*5*^ null, and *dfmr1*^*50M*^ null colabeled for pERK (green) and presynaptic membrane marker anti-HRP (magenta). pERK fluorescence shown as a heat map. NMJs shown without stimulation (basal, top) and with 90 mM [K^+^] HFS (high K^+^, bottom). Scale bar: 2.5 μm. **(E)** Quantified normalized basal presynaptic anti-pERK fluorescence for all 3 genotypes using one-way ANOVA and Tukey’s multiple comparisons. (**F)** Quantified normalized stimulated presynaptic anti-pERK fluorescence using one-way ANOVA and Tukey’s multiple comparisons. (**G**) Quantification of normalized presynaptic pERK levels in all 3 genotypes under basal and stimulated conditions using two-sided *t* tests. Scatter plots show all data points and mean ± SEM. *N =* number of animals (C) or NMJS (E-G). Significance: *p* > 0.05 (not significant, n.s.), *p* < 0.05 (*), *p* < 0.001 (**), *p* > 0.001 (***), and *p* < 0.0001 (****). The data underlying this figure can be found in S1 Data. *csw*, corkscrew; FMRP, Fragile X Mental Retardation Protein; GAPDH, glyceraldehyde 3-phosphate dehydrogenase; HFS, high-frequency stimulation; HRP, horseradish peroxidase; NMJ, neuromuscular junction; pERK, phosphorylated ERK; RIP, RNA-immunoprecipitation.

The FMRP negative translational regulator is well known to inhibit MAPK/ERK signaling in the regulation of synaptic function [13]. Moreover, high-throughput RNA sequencing from isolated crosslinking immunoprecipitation shows FMRP binds *csw* homolog *PTPN11/SHP2* mRNA [14]. Therefore, we hypothesized FMRP binds *csw* mRNA to negatively regulate translation upstream of MAPK/ERK signaling. To test this hypothesis, we first performed RNA-immunoprecipitation (RIP) studies with tagged FMRP::YFP from larval lysates using magnetic GFP-trap beads [47,48]. We used Tubby::GFP lysates as the RIP negative control, with α*-tubulin* (FMRP does not bind) as the internal negative control, and *futsch/MAP1B* (known FMRP target) as the internal positive control [17]. Immunoprecipitated mRNAs were reverse transcribed and tested with specific primers on 2% agarose gels. We next used western blots from larval ventral nerve cord (VNC)/brain lysates to test neuronal Csw protein levels with a characterized anti-Csw antibody [9]. Antibody specificity was confirmed with the *csw*^*5*^ null and protein levels compared between the genetic background control (*w*^*1118*^) and FXS disease model (*dfmr1* null mutants). To compare neuronal Csw protein levels in these different genotypes, we normalized to glyceraldehyde 3-phosphate dehydrogenase (GAPDH), a housekeeping gene that we confirmed is not regulated by Csw. Normalized quantification was done to compare neuronal Csw protein levels in the *w*^*1118*^ controls, *csw*^*5*^ null mutants, and *dfmr1*^*50M*^ null mutants. Representative RIP gels, western blots, and western blot quantified data are shown in Fig 6.

For the RIP analyses, *csw*, *futsch*, and α*-tubulin* mRNA bands are all present in both Tubby::GFP control and FMRP::YFP input lysates (Fig 6A). Immunoprecipitation pulls down *csw* mRNA from the FMRP::YFP third instar lysates, with no binding in the Tubby::GFP control (Fig 6A). Additionally, the positive control *futsch* mRNA is pulled down, but there is no detectable negative control α*-tubulin* mRNA. These results indicate FMRP binds *csw* mRNA, with the controls confirming binding interaction specificity. Based on this and above findings, we hypothesized FMRP partly inhibits NMJ synaptic transmission by suppressing Csw translation in neurons to decrease MAPK/ERK signaling. To test this hypothesis, western blot analyses were done to test Csw protein levels in larval brain/VNC lysates from controls (*w*^*1118*^), *csw*^*5*^, and *dfmr1*^*50M*^ null mutants. At the predicted molecular weight (100 kDa), there is a clear Csw band present in controls (Fig 6B). This band is undetectable in *csw*^*5*^ nulls, demonstrating specificity (Fig 6B). In the FXS disease model, there are clearly and consistently increased Csw protein levels in *dfmr1* null mutants (Fig 6B). Quantified comparisons normalized to GAPDH (*p* < 0.0001, ANOVA) show an increase in Csw levels in *dfmr1* nulls (1.55 ± 0.13) compared to controls (0.99 ± 0.029), which reveals a highly significant increase in the FXS disease model (*p* = 0.0008, Tukey’s, Fig 6C). There is slight background in *csw*^*5*^ (0.23 ± 0.06), which is very significantly decreased from controls (*p* < 0.0001, Tukey’s) and *dfmr1* mutants (*p* < 0.0001, Tukey’s; Fig 6C). Thus, *dfmr1* nulls have a strong increase in Csw levels in the larval neurons. Taken together, these findings show FMRP binds *csw* mRNA to negatively regulate Csw protein levels. We hypothesized this interaction negatively regulates presynaptic MAPK/ERK signaling.

### FMRP and Csw interact to inhibit presynaptic MAPK/ERK signaling and neurotransmission

We next set forth to test MAPK/ERK signaling within presynaptic boutons in order to begin investigating how FMRP and Csw interact to control presynaptic transmission. Elevated presynaptic pERK is well known to positively regulate neurotransmitter release function [49]. Based on this known role and our above studies, we hypothesized locally elevated pERK levels should occur in both *csw* and *dfmr1* null synaptic boutons. To test this hypothesis, we assayed NMJ terminals double-labeled with anti-pERK [50] and anti-horseradish peroxidase (HRP) to mark presynaptic bouton membranes. Using HRP to delineate presynaptic boutons, we measured pERK fluorescence intensity normalized to the genetic background control (*w*^*1118*^). Presynaptic pERK signaling is activity-dependent [51,52]. To test this function, we compared presynaptic pERK levels in the basal resting condition to stimulation with acute (10 minute) high [K^+^] depolarization (90 mM; [53,54]) in *w*^*1118*^ control, *dfmr1*^*50M*^ null mutant, and *csw*^*5*^ null mutant. We hypothesized that FMRP and Csw interact to inhibit presynaptic pERK signaling-dependent transmission strength. To test this hypothesis, we assayed the double *trans*-heterozygous *csw*^*5*^/+; *dfmr1*^*50M*^ /+ mutant compared to both single heterozygous mutants alone [28]. We first used TEVC recordings to measure stimulation evoked EJC responses and spontaneous mEJC release events. We then used pERK/HRP double-labeled imaging to measure the presynaptic pERK fluorescence intensity levels. Representative raw data of recordings and images as well as quantified results are shown in Fig 6.

Activated pERK is weakly detectable at control synapses under basal resting conditions (Fig 6D, top). In *w*^*1118*^ controls, pERK is localized at relatively higher levels in the presynaptic boutons, with lower levels of signaling in the adjacent muscle nuclei and very low sporadic levels throughout the muscle. Given the consistent presynaptic phenotypes above, we focused analyses on pERK signaling within presynaptic boutons. Compared to controls, both *csw* and *dfmr1* null mutants display consistently elevated pERK levels within the presynaptic boutons (Fig 6D, top), but with similar levels of pERK fluorescence in muscle compared to the controls. Similar results occur in *PTPN11* human patient mutants compared to driver controls (S9A Fig), with elevated pERK levels in all conditions (S9B Fig). This increased presynaptic pERK signaling and lack of postsynaptic changes is consistent with presynaptic perturbations in both *csw* and *dfmr1* null mutants. Quantification of the normalized pERK fluorescent intensity within the HRP-delineated presynaptic boutons shows very highly elevated levels in both the *csw* (1.85 ± 0.25, *n =* 15) and *dfmr1* (1.58 ± 0.13, *n* = 18) null mutants compared to controls (1.0 ± 0.12, *n* = 24), which is a significant increase (*p* = 0.001, one-way ANOVA; Fig 6E). When compared individually, there is no significant difference between *dfmr1* and *csw* mutants (*p* = 0.526, Tukey’s), showing both *csw* (*p* = 0.001, Tukey’s) and *dfmr1* (*p* = 0.024, Tukey’s; Fig 6E) nulls increase pERK signaling to a similar degree compared to controls. This elevated presynaptic pERK in both disease models fits our hypothesis that elevated MAPK/ERK signaling causes the increased presynaptic transmission in both disease models. Given the above changes in activity-dependent presynaptic function in *csw* null mutants, we next wanted to test whether pERK levels are dynamic and change with a stimulation challenge, and whether activity-dependent impairments occur in the two disease models.

When NMJs are strongly stimulated by acute depolarization (90 mM [K^+^] for 10 minutes), *w*^*1118*^ controls exhibit sharply increased presynaptic pERK levels compared to the basal resting condition (Fig 6D, bottom). Both *dfmr1* and *csw* nulls show smaller pERK level increases upon stimulation. This elevation shows pERK levels can be further increased in null mutants, indicating that the mechanism behind the increase is not exhausted under basal conditions or is controlled by other mechanisms beyond activity. Quantification of presynaptic pERK fluorescent intensity levels normalized to rest (*p* = 0.007, one-way ANOVA) shows pERK elevation in controls (1.68 ± 0.12, *n =* 21), *csw* nulls (2.44 ± 0.22, *n* = 15), and *dfmr1* (2.09 ± 0.14, *n =* 15) nulls, with *csw* exhibiting a significant elevation compared to controls (*p* = 0.005, Tukey’s; Fig 6F). When stimulated, pERK levels are similar in *csw* and *dfmr1* (*p* = 0.341, Tukey’s); however, *dfmr1* nulls are no longer significantly increased compared to controls (*p* = 0.192, Tukey’s; Fig 6F). To further assay activity-dependent changes, we directly compared the basal and stimulated pERK levels. Importantly, controls exhibit a significant activity-dependent presynaptic pERK increase when compared to rest (*p* = 0.0003, two-sided *t* test; Fig 6G). In contrast, *csw* nulls display only a trending elevation in stimulated pERK levels, without a significant increase from rest (*p* = 0.083, two-sided *t* test; Fig 6G). Likewise, *dfmr1* nulls display a reduced activity-dependent increase in stimulated presynaptic pERK levels compared to the basal condition, albeit still significant (*p* = 0.014, two-sided *t* test; Fig 6G). We conclude that the basal elevation in pERK levels in both disease models blunts further activation in response to stimulation. This activity-dependent defect correlates with the above impaired functional neurotransmission dynamics in response to stimulation. Based on the perturbed presynaptic pERK signaling in *csw* and *dfmr1* nulls, we hypothesized FMRP and Csw interact to inhibit synaptic MAPK/ERK signaling and transmission.

We therefore directly tested for this mechanism with *csw*/+; *dfmr1*/+ *trans*-heterozygotes. In TEVC recordings, these *trans*-heterozygotes show elevated neurotransmission compared to *w*^*1118*^ controls and both of the single heterozygotes (S10A Fig). Quantification reveals that the *csw*/+; *dfmr1*/+ *trans*-heterozygotes have higher EJC amplitudes (237.80 ± 7.5810 nA, *n =* 20) compared to *w*^*1118*^ controls (169.67 ± 8.1240 nA, *n* = 32), a significant increase (*p* < 0.0001, Dunnett’s; S10B Fig). In contrast, both *csw*/+ (199.10 ± 10.92 nA, *n* = 23) and *dfmr1*/+ (194.0 ± 11.36 nA, *n* = 18) heterozygotes display similar EJC amplitudes comparable to the *w*^*1118*^ control (S10A Fig), with no significant elevation (*p* = 0.19/0.058, Dunnett’s; S10B Fig). In mEJC recordings, double *csw*/+; *dfmr1*/+ *trans*-heterozygotes display a clear increase in mEJC frequency compared to both *w*^*1118*^ control and single heterozygotes (S10C Fig). Quantification shows *trans*-heterozygote mEJC frequency (2.60 ± 0.29 Hz, *n* = 16) elevated compared to *w*^*1118*^ (1.34 ± 0.15 Hz, *n* = 19), a significant increase (*p* = 0.0002, Dunn’s; S10D Fig). Both of the single heterozygotes, *csw*/+ (1.69 ± 0.19 Hz, *n* = 16) and *dfmr1*/+ (1.91 ± 0.26 Hz, *n* = 15), display a similar frequency comparable to *w*^*1118*^ control (S10C Fig), with no significant change (*p* = 0.428/0.151, Dunn’s; S10D Fig). There are no significant changes in the mEJC amplitudes (*p* = 0.855, Kruskal–Wallis; S10E Fig), confirming a presynaptic mechanism. Activated pERK labeling shows *csw*/+; *dfmr1*/+ *trans*-heterozygotes have elevated presynaptic signaling compared to *w*^*1118*^ control and the single heterozygotes (S10F Fig). Quantification shows increased presynaptic pERK fluorescence intensity in the trans-heterozygote (1.64 ± 0.11, *n* = 34) normalized to control (1.0 ± 0.07, *n* = 41), a significant elevation (*p* < 0.0001, Dunn’s; S10G Fig). Both of the single heterozygotes, *csw*/+ (1.02 ± 0.10, *n* = 31) and *dfmr1*/+ (1.23 ± 0.12, *n* = 34) have presynaptic pERK levels comparable to the control (S10F Fig), showing no significant change (*p* > 0.999/0.312, Dunn’s; S10G Fig). Taken together, these findings indicate FMRP and Csw interact to regulate presynaptic pERK signaling upstream of neurotransmitter release.

## Discussion

MAPK is well known to regulate activity-dependent signal transduction and synaptic plasticity within the nervous system [55]. Four MAPK families have been characterized, including extracellular signal-regulated kinase 1/2 (ERK1/2), ERK5, p38 MAPK, and the c-Jun N-terminal kinase (JNK; [56]). These families are activated similarly through an evolutionarily conserved cascade involving initial activation of GTPases (Ras/Rac) and a subsequent three-tiered protein kinase signaling system [57]. The best-characterized MAPK pathway, ERK1/2, has been extensively investigated within the nervous system, where ERK activation is very tightly regulated. Numerous neurological disease states display elevated ERK activity, including FXS, NS, and NSML, as well as neurodegenerative diseases such as Alzheimer’s and Parkinson’s disease [10,13,58]. Many studies have linked such elevated ERK signaling to cognitive deficits, particularly impairment of LTM consolidation. LTM requires spaced learning sessions during which ERK is activated and then decays in a temporal cycle. In *Drosophila* PTPN11/SHP2 homolog *csw* mutants, this ERK activation timing cycle is perturbed and LTM is disrupted [11]. Moreover, one of the targets of FMRP, a negative translational regulator, is *PTPN11/SHP2* mRNA [14], suggesting a potential link between the FXS and NS/NSML disease states. Based on the common ERK signaling up-regulation in these disorders, we hypothesized FMRP regulates Csw translation to modulate synaptic ERK levels to control neurotransmission strength and functional plasticity.

This hypothesis provides the first proposed mechanistic connection between NS, NSML, and FXS disease conditions, through an ERK phosphorylation (pERK) signaling defect in presynaptic boutons. pERK is known to activate presynaptic function, with short-term roles in the control of neurotransmission strength and activity-dependent plasticity [49,59], and longer-term nuclear translocation roles [57]. In the *Drosophila* NS/NSML disease models of *csw* LoF and GoF, we began with synaptic transmission assays at the NMJ glutamatergic synapse [32]. We also tested human patient *PTPN11/SHP2* mutations to confirm functional requirements [12]. Our work reveals that all LoF/GoF mutations elevate neurotransmission strength, indicating that Csw/SHP2 is involved in inhibiting glutamatergic signaling. Consistently, previous *Drosophila* NS and NSML model studies also show that LoF and GoF mutations phenocopy one another, with a correlation to hyperactivated pERK signal transduction in both conditions [9,10]. Moreover, the *Drosophila* FXS disease model similarly increases NMJ glutamatergic synaptic transmission [17], consistent with the FMRP mechanistic intersection. Localized pERK signaling occurs on both pre- and postsynaptic sides [60,61], so we next used cell-targeted *csw* RNAi and measured spontaneous vesicle fusion events to separate these requirements. Our work reveals Csw/SHP2 has only a neuronal role in the regulation of presynaptic transmission. There is no detectable postsynaptic function. This new presynaptic Csw/SHP2 role is consistent with the abundant evidence for both MAPK/ERK and FMRP involvement in modulating glutamatergic release mechanisms.

Presynaptic vesicle fusion is a major determinant of neurotransmission strength, maintained functional resilience during strong demand, and activity-dependent plasticity [62]. HFS trains cause the transient activation of pERK signaling in presynaptic terminals [51], correlating with increased vesicle fusion. To test if Csw/SHP2 similarly regulates glutamate release, we performed HFS synaptic depression assays to discover that all mutants have increased transmission resiliency under conditions of heightened demand [34], with elevated glutamate release from presynaptic boutons. This role is consistent with activity-dependent presynaptic MAPK/ERK signaling driving greater presynaptic glutamate release by modulating the accessible number of synaptic vesicles available for fusion in the RRP [19]. Importantly, the mouse FXS disease model displays similar decreased short-term depression due to enhanced presynaptic glutamate release, also via up-regulation of the RRP without a change in PPR fusion [20]. The MAPK/ERK-dependent phosphorylation of presynaptic targets is likewise known to increase short-term plasticity, and blockade of this signaling process has been shown to strongly impair facilitation, maintained augmentation, and PTP [51,63]. Our results show that all three forms of synaptic plasticity are impaired in *csw* null and *PTPN11*^*N308D*^ GoF animals, which both show decreased facilitation, augmentation, and PTP, consistent with other LoF/GoF phenocopy. We hypothesize that these plasticity defects correlate to the already increased basal transmission levels that cause a decrease in range for enhancement from presynaptic pERK activation, leading to a “ceiling effect” on presynaptic function. This predicts neurotransmission defects are linked to causal changes in presynaptic MAPK/ERK signaling.

Both NS and NSML disease states exhibit elevated MAPK/ERK signaling [10], but there is heterogeneity in pERK activation levels and multiple pathways involved [12]. To confirm the neurotransmission increase is due to elevated MAPK/ERK signaling, we inhibited this pathway with both Trametinib and Vorinostat, two drugs well characterized to decrease pERK signaling [46,64]. With drug treatments, the elevated neurotransmission in *csw* and *PTPN11* mutants is restored to levels comparable to control animals, indicating that the elevated MAPK/ERK signaling is responsible for the heightened presynaptic function. This test does not rule out the possibility of other disrupted signaling pathways that may influence MAPK/ERK signaling, but does prove MAPK/ERK signaling is the cause of the elevated neurotransmission. The next task was to explore the new activity-dependent mechanism controlling this presynaptic Csw/SHP2 function. As previously discussed, NS, NSML, and FXS models/patients all display striking similarities in up-regulated MAPK/ERK signaling, synaptic phenotypes, and LTM impairments [17,18,20]. Moreover, RNA-binding FMRP is well characterized as an activity-dependent negative translational regulator of presynaptic mRNA targets [65]. Consistently, we find that *Drosophila* FMRP binds *csw* mRNA, as suggested in a mouse FMRP screen indicating *PTPN11/SHP2* binding [14]. Additionally, we find neuronal Csw protein levels are elevated in the FXS disease model (*dfmr1* null), consistent with the predicted FMRP translational repression [66]. Finally, we find that presynaptic pERK signaling is increased in both *dfmr1* and *csw* null mutants and that normal activity-dependent elevation in pERK signaling is impaired in both disease model conditions. The pERK enhancement levels are slightly different, but this to likely due to the relative effect of the two nulls on pERK signaling. The heightened basal presynaptic pERK signaling and repressed activity-dependent pERK signaling suggests that FMRP and Csw interact to modulate presynaptic glutamatergic neurotransmission.

One genetic test for pathway interaction employs nonallelic noncomplementation [67], which demonstrates that the two gene products operate within a common mechanism, in this case, the up-regulation of MAPK signaling [28]. Both *dfmr1* and *csw* null mutants display elevated presynaptic neurotransmission with an increased probability of presynaptic glutamate release [17], and *trans*-heterozygous *dfmr1*/+; *csw*/+ double mutants recapitulate both functional phenotypes. Importantly, both the *dfmr1* and *csw*^*5*^ single heterozygous mutants do not display any phenotypes, despite the NSML autosomal dominant disease state. Similarly, Csw/PTPN11 overexpression does not cause any phenotypes, suggesting a change in the FXS background causes the elevated MAPK/ERK presynaptic signaling. These genetic tests indicate that FMRP and Csw/SHP2 act together to inhibit pERK signaling and presynaptic glutamate release. We propose the mechanism of mRNA-binding FMRP acting canonically as a negative translational regulator of Csw/SHP2 expression [68]. Both the *dfmr1* and *csw* null mutants display elevated MAPK/ERK signaling as indicated by pERK production [56], and we demonstrate here pERK elevation in presynaptic boutons. Consistent with a common mechanism, *trans*-heterozygous *csw*/+; *dfmr1*/+ mutants recapitulate this heightened presynaptic pERK signaling. We propose the mechanism of FMRP working through Csw/SHP2 phosphatase enzymatic activity to inhibit presynaptic pERK production. Given that MAPK/ERK signaling is well established to modulate presynaptic glutamatergic release [49], we suggest heightened presynaptic pERK signaling causes elevated glutamate release probability. We demonstrate this causal link with pharmacological treatments that block pERK production [45], which act to restore normal glutamatergic synaptic signaling in the disease model animals.

In conclusion, we note that there are important differences between FXS and NS/NSML disease models. Previous FXS model work has shown increased NMJ architecture and mEJC amplitudes in *dfmr1* nulls [17], which are absent in NS/NSML model *csw/PTPN11* mutants. FXS is a very complex disease state with many proteins misregulated [17], and there was never an expectation that all FXS phenotypes would be recapitulated in *csw/PTPN11* mutants, especially for the unrelated postsynaptic changes. Nevertheless, the presynaptic parallels are striking. The mouse FXS model exhibits decreased short-term depression with no change in PPR, but an increase in RRP [20], matching the *Drosophila* results shown here. Interestingly, these phenotypes match closer than mouse *H-ras*^*G12V*^ mutants with increased pERK signaling, which exhibit enhanced short-term synaptic plasticity [19], compared to the depressed plasticity shown here. Thus, although both basal transmission strength and functional plasticity properties are dependent on presynaptic MAPK/ERK signaling, there are likely other intersecting regulatory pathways. Moreover, FMRP and Csw/SHP2 could interact via multiple different mechanisms to regulate presynaptic MAPK/ERK signaling, and the elevated neurotransmission in the disease state models may not be completely dependent on presynaptic MAPK/ERK signaling. In the FXS model, Csw/SHP2 is both up-regulated and hyperactivated, and the mechanism of this activation is unknown. One possibility is decreased MAPK/ERK negative regulation, via other factors like Neurofibromin-1, which could further increase MAPK/ERK signaling [69,70]. Another possibility is that neuronal activity up-regulates and then activates Csw/SHP2 via two parallel mechanisms to increase MAPK/ERK signaling [71,72]. We have previously uncovered several other genetic mutants that likewise elevate neurotransmission and depress short-term plasticity [28,73–75], which are also candidates for furthering our understanding in future studies. The possibility for a more extensive interactive molecular network is exciting, but it can currently only be concluded that FMRP and Csw/SHP2 both control MAPK/ERK signaling and modulate neurotransmission. This presynaptic mechanism connects the previously unlinked disorders of NS, NSML, and FXS, suggesting common therapeutic targets and new treatment avenues.

## Materials and methods

### *Drosophila* genetics

All the *Drosophila* stocks were reared on standard cornmeal/agar/molasses food at 25°C within 12-hour light/dark cycling incubators. All animals were reared to the wandering third instar stage for all experiments, with all genotypes and RNAi lines confirmed with a combination of transgenically marked balancer chromosomes, western blots, and sequencing. Due to the *corkscrew* gene being on the X chromosome, all experiments utilizing *csw*^*5*^ mutants were conducted using males only, whereas all the *trans*-heterozygous experiments were done using females only. All the other experiments were done on both of the sexes (males and females together). The two genetic background controls were *w*^*1118*^ and the TRiP RNAi third chromosome background control [31]. The *dfmr1*^*50M*^ null mutant [17], *csw*^*5*^ null mutant [24], and the transgenic lines UAS-*csw*^*WT*^ and UAS-*csw* RNAi [25,30] are all available from the *Drosophila* Bloomington Stock Center (BDSC; Indiana University, Bloomington, IN, USA). The UAS-*csw*^*A72S*^ line [9] was obtained as a kind gift from Dr. Mario Rafael Pagani (Department of Physiology and Biophysics, School of Medicine, National Scientific and Technical Research Council, University of Buenos Aires, Buenos Aires, Argentina). All patient-derived UAS-*PTPN11* mutant lines [12] were obtained as a kind gift from Dr. Tirtha Das (Department of Cell, Developmental, and Regenerative Biology, Icahn School of Medicine at Mount Sinai, New York, NY, USA). Transgenic studies were performed with neural-specific *elav-*Gal4 [76], muscle-specific *24B*-Gal4 [77], and ubiquitous *daughterless UH1*-Gal4 [78] driver lines, all obtained from BDSC. The genetic and transgenic lines used in this study are summarized below in Table 1:

**Table 1:** *Drosophila* mutant and transgenic lines used in this study.

Line	Provider	Reference
*csw* ^*5*^	BDSC 23874	(Perrimon et al., 1985) [24]
UAS-*csw*^*WT*^	BDSC 23878	(Hamlet and Perkins, 2001) [25]
UAS-*csw*^*A72S*^	Dr. Mario Rafael Pagani	(Oishi et al., 2006) [9]
UAS-*PTPN11*^*R498W*^	Dr. Tirtha Das	(Das et al., 2021) [12]
UAS-*PTPN11*^*Y279C*^	Dr. Tirtha Das	(Das et al., 2021) [12]
UAS-*PTPN11*^*Q510E*^	Dr. Tirtha Das	(Das et al., 2021) [12]
UAS-*PTPN11*^*Q510P*^	Dr. Tirtha Das	(Das et al., 2021) [12]
UAS-*csw* RNAi	BDSC 33619	(Ni et al., 2011) [30]
TriP third RNAi Ctl	BDSC 36303	(Perkins et al., 2015) [31]
*dfmr1* ^*50M*^	BDSC 6930	(Zhang et al., 2001) [17]
*UAS-YFP-dfmr1*	Dr. Daniela Zarnescu	(Cziko et al., 2009) [47]

### Synaptic electrophysiology

Wandering third instar dissections and TEVC recordings were done at 18°C in physiological saline (in mM): 128 NaCl, 2 KCl, 4 MgCl_2_, 1.0 CaCl_2_, 70 sucrose, and 5 HEPES (pH 7.2). Staged larvae were dissected longitudinally along the dorsal midline, the internal organs removed, and the body walls glued down (Vetbond, 3M). Peripheral motor nerves were cut at the base of the VNC. Dissected preparations were imaged with a Zeiss 40× water-immersion objective on a Zeiss Axioskop microscope. Muscle 6 in abdominal segments 3 to 4 was impaled with two intracellular electrodes (1 mm outer diameter borosilicate capillaries; World Precision Instruments, 1B100F-4) of approximately 15 MΩ resistance when filled with 3M KCl. The muscles were clamped at −60 mV using an Axoclamp-2B amplifier (Axon Instruments). For evoked EJC recordings, the motor nerve was stimulated with a fire-polished suction electrode using 0.5 ms suprathreshold voltage stimuli at 0.2 Hz from a Grass S88 stimulator. Nerve stimulation–evoked EJC recordings were filtered at 2 kHz. To quantify EJC amplitude, 10 consecutive traces were averaged, and the average peak value recorded. Spontaneous mEJC recordings were made in continuous 2-minute sessions and low-pass filtered at 200 Hz. Synaptic depression experiments were performed using the above EJC recording protocol for 1 minute to establish baseline, followed by a 20-Hz HFS train for 5 minutes at the same suprathreshold voltage. RRP size was estimated by dividing the cumulative EJC amplitudes during the first 100 responses to 20 Hz stimulation by the mean mEJC amplitudes. Due to these analyses being at 20 Hz, RRP size is likely underestimated. All synaptic plasticity experiments were performed in 0.2 mM Ca^2+^ using 10 Hz stimulation trains for 1 minute, followed by 0.2 Hz recordings. All EJC responses within a 1-second bin were averaged, and the average value normalized to the basal EJC amplitude for each animal. Clampex 9.0 was used for all data acquisition, and Clampfit 10.6 was used for all data analyses (Axon Instruments).

### Drug treatments

Two drugs known to inhibit pERK production (Trametinib and Vorinostat) were used by feeding as published previously [12,45,46]. Both Trametinib (Cell Signaling, 62206S) and Vorinostat (Cell Signaling, 12520S) were dissolved in dimethylsulfoxide (DMSO; Fisher, 67-68-5) at 15 mM and 20 mM, respectively, to create stock solutions. Both drugs were then added to *Drosophila* food yeast paste and in the standard cornmeal/agar/molasses food in the final concentrations of 0.5 mM (Trametinib) and 1 mM (Vorinostat). *Drosophila* were induced to lay eggs on selection apple juice plates with drugged yeast paste food. Hatching first instars were selected and placed in standard vials containing Trametinib, Vorinostat, or control food with DMSO only. Larvae were reared in a 12-hour light/dark cycling incubators at 25°C and then collected as wandering third instars for TEVC studies.

### RNA immunoprecipitation

Wandering third instars (20 larvae) of each genotype (UH1>FMRP-YFP or Tubulin-GFP) were homogenized in 200 μL of RNase-free lysis buffer (20 mM HEPES, 100 mM NaCl, 2.5 mM EDTA, 0.05% (v/v) Triton X-100, 5% (v/v) glycerol) with 1% *β*-mercaptoethanol 1× protease inhibitor cocktail (complete mini EDTA-free Tablets, Sigma, 11836170001) and 400U RNase inhibitor (Applied Biosystems, N8080119). The supernatant was collected and diluted to 300 μL to reduce nonspecific binding. Next, the samples were incubated with GFP-trap coupled magnetic agarose beads (Chromotek, GTMA20) for 3 hours at 4°C. The bound beads were washed with lysis buffer (3X, 10 minutes). The bound RNA was purified by incubating the bead-protein-RNA conjugates with a 500-μL TRIzol and chloroform mixture (Ambion, 15596026) for 10 minutes at RT, followed by centrifugation. To precipitate RNA, glycogen (1 μL) and 2-propenol (250 μL) were added to the isolated aqueous layer. Finally, the precipitated RNA was reverse transcribed into single-strand cDNA using the SuperScript VILO cDNA synthesis kit (Thermo Fisher, 11754050) and then subjected to primer-specific PCR, with 2% agarose gels used to analyze the PCR products. All primers used in this study are summarized above in Table 2.

**Table 2:** Primers used for RNA immunoprecipitation. The length of PCR products is approximately 200 bp.

Primer	Sequence
*corkscrew* (forward)	CTACCGCAACATATTGCCATACGAC
*corkscrew* (reverse)	CTGCACGCACGTCTTGTTTT
*futsch* (forward)	TTCCTGGATATTGCAGGACGG
*futsch* (reverse)	CTCGGGCAATGTGTGCCATA
α*-tubulin* (forward)	ATTTACCCAGCACCACAAGTGT
α*-tubulin* (reverse)	GGCGATTGAGATTCATGTAGGTGG

### Western blots

Wandering third instar VNCs from 20 larvae were homogenized in 100 μL of lysis buffer (20 mM HEPES, 10 mM EDTA, 100 mM KCl, 0.1% (v/v) Triton X-100, 5% (v/v) glycerol) with a protease inhibitor cocktail (Roche, 04693132001) combined with a protease and phosphatase inhibitor cocktail (Abcam, ab201119). All samples were then sonicated and run in 4% to 15% Mini-PROTEAN TGX Stain-Free Precast Gels (BioRad, 4568083) alongside Precision Plus Protein all blue prestained protein standards (BioRad, 1610373). Next, total protein was transferred to PVDF membranes using a Trans-Blot Turbo system (BioRad). After transfer, the membrane was blocked by TBS intercept blocking buffer (LiCOR, 927–60000) for 1 hour at RT. The blocked membranes were incubated with primary antibodies overnight at 4°C. Antibodies used include rabbit anti-Csw (Lizabeth Perkins, F1088, 1:500) and goat anti-GAPDH (Abcam, ab157157, 1:2,000). The membrane was washed with Tris-buffer saline with 0.1% Tween-20 (TBST) and then incubated with secondary antibodies for 40 minutes at RT. Secondary antibodies used include Alexa Fluor 680 donkey anti-goat (Invitrogen, A21084, 1:10,000) and Alexa Fluor 800 goat anti-rabbit (Invitrogen, A32735, 1:10,000). After washing with TBST (3X, 10 minutes), the membranes were imaged using the Li-COR Odyssey CLx system.

### Immunocytochemistry imaging

Wandering third instars were dissected in physiological saline (see above) and fixed in 4% paraformaldehyde (EMS, 15714) diluted in PBS (Corning, 46–013-CM) for 10 minutes at RT. Preparations were then washed and permeabilized in PBS containing 0.2% Triton X-100 and 1% bovine serum albumin (BSA; 3X, 10 minutes), followed by blocking for 30 minutes at RT in the same solution. Preparations were incubated with primary antibodies overnight at 4°C. Primary antibodies used included rabbit anti-pERK1/ERK2 (Thr185, Tyr187) polyclonal antibody (Thermo Fisher, 44-680G, 1:100), goat Cy3-conjugated anti-HRP (Jackson ImmunoResearch, 123–165–021, 1:200), and goat 488-conjugated anti-HRP (Jackson ImmunoResearch, 123–545–021, 1:200). Preparations were washed (3X, 10 minutes) and then incubated with secondary antibodies for 2 hours at RT. Secondary antibodies used included: donkey 488 anti-rabbit (Invitrogen, A21206) and donkey 555 anti-rabbit (Invitrogen, A31572). Preparations were washed (3X, 10 minutes) and then mounted in Fluoromount G (Electron Microscopy Sciences) onto 25 × 75 × 1 mm slides (Fisher Scientific, 12–544–2) with a 22 × 22–1 coverslip (Thermo Fisher Scientific, 12–542-B) sealed with clear nail polish (Sally Hansen). All NMJ imaging was performed using a Zeiss LSM 510 META laser-scanning confocal microscope, with images projected in Zen (Zeiss) and analyzed using ImageJ (NIH open source). All NMJ intensity measurements were made with HRP signal-delineated *z*-stack areas of maximum projection using ImageJ threshold and wand-tracing tools.

### Statistical analyses

All statistics were performed using GraphPad Prism software (v9.0). Data sets were subject to normality tests, with D’Agostino–Pearson tests utilized if *n* > 10 and Shapiro–Wilk tests if *n* < 10. With normal data, ROUT outlier tests with Q set to 1% were run, followed by either two-tailed Student *t* tests for two-way comparison with 95% confidence (2 data sets) or a one-way ANOVA followed by either a Tukey’s multiple comparison test (3+ data sets, comparing all samples) or a Dunnett’s multiple comparison test (3+ data sets, comparing to control). If data were not normal, Mann–Whitney tests (2 data sets) or Kruskal–Wallis followed by a Dunn’s multiple comparisons tests (3+ data sets) were performed. In order to fully capture changes in the datasets for experiments containing time courses, nonlinear regressions were performed followed by F extra sum of squares tests to determine if the curves were significantly different. All figures show all individual data points as well as mean ± SEM, with significance displayed as *p* ≤ 0.05 (*), *p* ≤ 0.01 (**), *p* ≤ 0.001 (***), *p* ≤ 0.0001 (****), and *p* > 0.05 (not significant; n.s.).

## Supporting information

S1 Fig NMJ architecture is unchanged in *csw* null and GoF mutants. **(A)** Representative NMJ images of the *w*^*1118*^ genetic background control, *csw*^*5*^ null mutant, *UH1*-Gal4/*w*^*1118*^ transgenic driver control, and *csw*^*A72S*^ GoF mutant (*UH1*-Gal4>*csw*^*A72S*^) colabeled for presynaptic membrane marker anti-HRP (magenta) and postsynaptic scaffold DLG (green). Scale bar: 10 μm. (**B)** Quantification of muscle length for all 4 genotypes using two-sided *t* tests. (**C)** Quantification of NMJ area for all 4 genotypes using Mann–Whitney tests. (**D)** Quantification of NMJ branch number for all 4 genotypes using Mann–Whitney tests. (**E)** Quantification of NMJ synaptic bouton number for all 4 genotypes using Mann–Whitney tests. Scatter plots show all the individual data points as well as mean ± SEM. *N =* number of NMJs. Significance: *p* > 0.05 (not significant, n.s.). The data underlying this figure can be found in S1 Data. *csw*, corkscrew; DLG, Discs Large; GoF, gain-of-function; HRP, horseradish peroxidase; NMJ, neuromuscular junction.
(TIF)

S2 Fig Synapse number is unchanged in *csw* null and GoF mutants. **(A)** Representative NMJ images of the *w*^*1118*^ genetic background control, *csw*^*5*^ null mutant, *UH1*-Gal4/*w*^*1118*^ transgenic driver control, and *csw*^*A72S*^ GoF mutant (*UH1*-Gal4>*csw*^*A72S*^) colabeled for presynaptic membrane marker anti-HRP (blue), active zone marker Brp (magenta), and postsynaptic GluRIIC (green). Scale bar: 2.5 μm. (**B)** Quantification of Brp puncta density for all 4 genotypes using two-sided *t* test/Mann–Whitney tests. (**C)** Quantification of GluRIIC puncta density for all 4 genotypes using two-sided *t* tests. (**D)** Quantification of the Brp:GluRIIC puncta ratio for all 4 genotypes using two-sided *t* test/Mann–Whitney tests. Scatter plots show all the individual data points as well as mean ± SEM. *N* = number of NMJs. Significance: *p* > 0.05 (not significant, n.s.). The data underlying this figure can be found in S1 Data. Brp, Bruchpilot; *csw*, corkscrew; GluRIIC, glutamate receptor IIC; GoF, gain-of-function; HRP, horseradish peroxidase; NMJ, neuromuscular junction.
(TIF)

S3 Fig Wild-type *Csw/PTPN11* expression restores neurotransmission in mutants. **(A)** Representative EJC traces for the *csw*^*5*^ null mutant rescued via expression of *csw*^*WT*^ (*csw*^*5*^ *UH1*-Gal4>*csw*^*WT*^) and transgenic driver control (*UH1*-Gal4/*w*^*1118*^) showing 10 superimposed responses (1.0 mM Ca^2^). (**B)** Quantification of the mean EJC amplitudes using a two-sided *t* test. (**C)** Representative EJC traces for the wild-type *PTPN11* (*UH1*-Gal4>*PTPN11*^*WT*^) and transgenic driver control (*UH1*-Gal4/*w*^*1118*^) showing 10 superimposed evoked synaptic responses (1.0 mM Ca^2^). (**D)** Quantification of the mean EJC amplitudes using a two-sided *t* test. Scatter plots show all the individual data points as well as mean ± SEM. *N =* number of NMJs. Significance: *p* > 0.05 (not significant, n.s.). The data underlying this figure can be found in S1 Data. *Csw*, corkscrew; EJC, excitatory junction current; NMJ, neuromuscular junction; *PTPN11*, protein tyrosine phosphatase non-receptor type 11.
(TIF)

S4 Fig Neuronal *csw* RNAi knockdown increases spontaneous fusion frequency. **(A)** Representative mEJC traces (1.0 mM Ca^+2^) in driver control (*UH1*-Gal4/TRiP control, top) and *UH1*-Gal4>*csw* RNAi (bottom). (**B)** Quantification of the mEJC frequency using a two-sided *t* test. (**C)** Quantification of mEJC amplitude using a Mann–Whitney test. (**D)** Representative mEJC traces (1.0 mM Ca^+2^) in driver control (*elav*-Gal4/TRiP control, top) and neuronal *elav*-Gal4>*csw* RNAi (bottom). (**E)** Quantification of the mEJC frequency using a Mann–Whitney test. (**F)** Quantification of the mEJC amplitude using a Mann–Whitney test. (**G)** Representative mEJC traces (1.0 mM Ca^+2^) in driver control (*24B*-Gal4/TRiP, top) and muscle *24B*-Gal4>*csw* RNAi (bottom). (**H)** Quantification of the mEJC frequency using a two-sided *t* test. (**I)** Quantification of the mEJC amplitude using two-sided *t* test. Scatter plots show all the individual data points as well as mean ± SEM. *N =* number of NMJs. Significance: *p* > 0.05 (not significant, n.s.), *p* < 0.05 (*), and *p* > 0.001 (***). The data underlying this figure can be found in S1 Data. *csw*, corkscrew; mEJC, miniature EJC; NMJ, neuromuscular junction; RNAi, RNA interference.
(TIF)

S5 Fig All *csw*/*PTPN11* mutants exhibit increased synaptic quantal content release. The quantal content at each NMJ was calculated by dividing the evoked EJC traces by the mean mEJC amplitude. (**A)** Quantification of the quantal content of both the *csw/PTPN11* null and GoF mutants using two-sided *t* tests. (**B)** Quantification of the quantal content of *csw RNAi* ubiquitous (*UH1*), neuronal (*elav*), and muscle (*24B*) lines compared to their matched transgenic driver controls using two-sided *t* tests. Scatter plots show all the individual data points as well as mean ± SEM. *N =* number of NMJs. Significance: *p* > 0.05 (not significant, n.s.), *p* < 0.05 (*), *p* > 0.001 (***), and *p* < 0.0001 (****). The data underlying this figure can be found in S1 Data. *Csw*, corkscrew; EJC, excitatory junction current; GoF, gain-of-function; mEJC, miniature EJC; NMJ, neuromuscular junction; *PTPN11*, protein tyrosine phosphatase non-receptor type 11; *RNAi*, RNA interference.
(TIF)

S6 Fig Neuronal NSML model *PTPN11* mutants exhibit elevated presynaptic function. **(A)** Representative EJC traces for the transgenic driver control (*elav*-Gal4/*w*^*1118*^), and *PTPN11* patient mutants *PTPN11*^*Q510E*^ (*elav*-Gal4>*PTPN11*^*Q510E*^) and *PTPN11*^*Q510P*^ (*elav*-Gal4>*PTPN11*^*Q510P*^) showing 10 superimposed evoked synaptic responses (1.0 mM Ca^2+^). (**B)** Quantification of the mean EJC amplitudes in all 3 genotypes using one-way ANOVA and Tukey’s multiple comparisons. (**C)** Representative mEJC traces (1.0 mM Ca^2+^) in above driver control (top), *PTPN11*^*Q510E*^ (middle), and *PTPN11*^*Q510P*^ (bottom). (**D)** Quantification of the mEJC frequency using one-way ANOVA and Tukey’s multiple comparisons. (**E)** Quantification of mEJC amplitude using a Kruskal–Wallis test. (**F)** Quantification of quantal content using one-way ANOVA and Tukey’s multiple comparisons. Scatter plots show all the individual data points as well as mean ± SEM. *N =* number of NMJs. Significance: *p* > 0.05 (not significant, n.s.), *p* < 0.05 (*), *p* < 0.001 (**), and *p* > 0.001 (***). The data underlying this figure can be found in S1 Data. EJC, excitatory junction current; mEJC, miniature EJC; NMJ, neuromuscular junction; NSML, NS with multiple lentigines; *PTPN11*, protein tyrosine phosphatase non-receptor type 11.
(TIF)

S7 Fig HFS transmission depression ameliorated in *csw* nulls. Prolonged HFS at 20 Hz (1 mM Ca^+2^) drives progressive synaptic amplitude depression over several minutes of continuous recording. (**A)** Representative evoked nerve-stimulated EJC traces at the basal frequency (t = 0) and indicated time points during the HFS train for the genetic background control (*w*^*1118*^, top) and the *csw* null mutant (*csw*^*5*^, bottom). (**B)** Quantification of normalized EJC amplitudes at the indicated time points during the HFS train using two-sided *t* tests. Scatter plots show all data points and mean ± SEM. *N* = number of NMJs. Significance: *p* < 0.05 (*), *p* < 0.001 (**), and *p* < 0.001 (***). The data underlying this figure can be found in S1 Data. *csw*, corkscrew; EJC, excitatory junction current; HFS, high-frequency stimulation; NMJ, neuromuscular junction.
(TIF)

S8 Fig Reducing ERK signaling restores NS model *PTPN11* synaptic function. TEVC recordings with and without the pERK inhibiting drug Trametinib comparing the driver control (*elav-Gal4/w*^*1118*^) and NS GoF patient mutant (*elav-Gal4>PTPN11*^*N308D*^). (**A)** Representative EJC traces showing 10 superimposed responses (1.0 mM Ca^+2^) comparing the control (left) and *PTPN11*^*N308D*^ mutant (right), with and without Trametinib. (**B)** Quantification of mean EJC amplitudes for all 4 conditions using one-way ANOVA and Tukey’s multiple comparisons. Scatter plots show all the data points and the mean ± SEM. *N* = number of NMJs. Significance: *p* > 0.05 (not significant, n.s.) and *p* < 0.05 (*). The data underlying this figure can be found in S1 Data. EJC, excitatory junction current; ERK, extracellular signal-regulated kinase; GoF, gain-of-function; NMJ, neuromuscular junction; NS, Noonan syndrome; pERK, phosphorylated ERK; *PTPN11*, protein tyrosine phosphatase non-receptor type 11; TEVC, two-electrode voltage-clamp.
(TIF)

S9 Fig *PTPN11* LoF and GoF mutants exhibit elevated presynaptic pERK levels. **(A)** Representative NMJ images of the driver control (*UH1-*Gal4*/w*^*1118*^, top left), the GoF mutant (*elav>PTPN11*^*N308D*^; top right), and two LoF mutants (*UH1-*Gal4*>PTPN11*^*Q510E*^, bottom left, and *UH1-Gal4>PTPN11*^*Q510P*^; bottom right) colabeled for presynaptic membrane marker anti-HRP (magenta) and pERK (green). Scale bar: 2.5 μm. **(B)** Quantified presynaptic anti-pERK fluorescence for all 5 genotypes using a two sided *t* test (*PTPN11*^*N308D*^) and one-way ANOVA and Tukey’s multiple comparisons (*PTPN11*^*Q510E*^/ *PTPN11*^*Q510P*^). Scatter plots show all data points and mean ± SEM. *N* = number of NMJs. Significance: *p* < 0.001 (**), *p* > 0.001 (***), and *p* < 0.0001 (****). The data underlying this figure can be found in S1 Data. GoF, gain-of-function; HRP, horseradish peroxidase; LoF, loss-of-function; NMJ, neuromuscular junction; pERK, phosphorylated ERK; *PTPN11*, protein tyrosine phosphatase non-receptor type 11.
(TIF)

S10 Fig *Trans*-heterozygous *csw*/+; *dfmr1*/+ recapitulate disease model phenotypes. **(A)** Representative evoked EJC traces showing 10 superimposed TEVC recordings in background control (*w*^*1118*^), single heterozygotes (*csw*^*5*^/+ and *dfmr1*^*50M*^/+), and the *trans*-heterozygote (*csw*^*5*^/+; *dfmr1*^*50M*^/+). (**B)** Quantification of mean EJC amplitudes for all 4 genotypes using one-way ANOVA and Dunnett’s multiple comparisons. (**C)** Representative mEJC traces from the same 4 genotypes. (**D)** Quantification of mEJC frequency for all 4 genotypes using Kruskal–Wallis and Dunn’s multiple comparisons. (**E)** Quantification of mEJC amplitude for all 4 genotypes using Kruskal–Wallis. (**F)** Representative NMJ images from the same 4 genotypes colabeled for anti-pERK (green) and presynaptic membrane anti-HRP (magenta). pERK also shown as a heat map. Scale bar: 2.5 μm. (**G)** Quantification of normalized synaptic pERK fluorescence for all 4 genotypes using Kruskal–Wallis and Dunn’s multiple comparison tests. Scatter plots show all data points and the mean ± SEM. *N* = number of NMJs. Significance: *p* > 0.05 (not significant, n.s.), *p* < 0.001 (**), *p* > 0.001 (***), and *p* < 0.0001 (****). The data underlying this figure can be found in S1 Data. EJC, excitatory junction current; HRP, horseradish peroxidase; mEJC, miniature EJC; NMJ, neuromuscular junction; pERK, phosphorylated ERK; TEVC, two-electrode voltage-clamp.
(TIF)

S1 Data Excel document detailing raw data for all analyses. (XLSX)

S1 Raw Images Original uncropped gel and blot of Fig 6. (TIF)

## Abbreviations

BSA: bovine serum albumin
csw: corkscrew
dfmr1: *Drosophila* fragile X mental retardation 1
DMSO: dimethylsulfoxide
EJC: excitatory junction current
ERK: extracellular signal-regulated kinase
FMRP: Fragile X Mental Retardation Protein
FXS: Fragile X syndrome
GAPDH: glyceraldehyde 3-phosphate dehydrogenase
GoF: gain-of-function
HFS: high-frequency stimulation
HRP: horseradish peroxidase
JNK: c-Jun N-terminal kinase
LoF: loss-of-function
LTM: long-term memory
MAPK: mitogen-activated protein kinase
mEJC: miniature excitatory junction current
NMJ: neuromuscular junction
NS: Noonan syndrome
NSML: NS with multiple lentigines
pERK: phosphorylated ERK
PPR: paired-pulse ratio
PTP: post-tetanic potentiation
PTPN11: protein tyrosine phosphatase non-receptor type 11
RIP: RNA-immunoprecipitation
RNAi: RNA interference
RRP: readily releasable pool
SHP2: SH2 domain-containing protein tyrosine phosphatase-2
TBST: Tris-buffer saline with 0.1% Tween-20
TEVC: two-electrode voltage-clamp
TRiP: Transgenic RNAi Project
VNC: ventral nerve cord
