# Supplementary figures and images for: FMRP activity and control of Csw/SHP2 translation regulate MAPK-dependent synaptic transmission
Source: PLoS Biol. 2023 Jan 26;21(1):e3001969. doi: 10.1371/journal.pbio.3001969 (PMC9879533; doi:10.1371/journal.pbio.3001969)

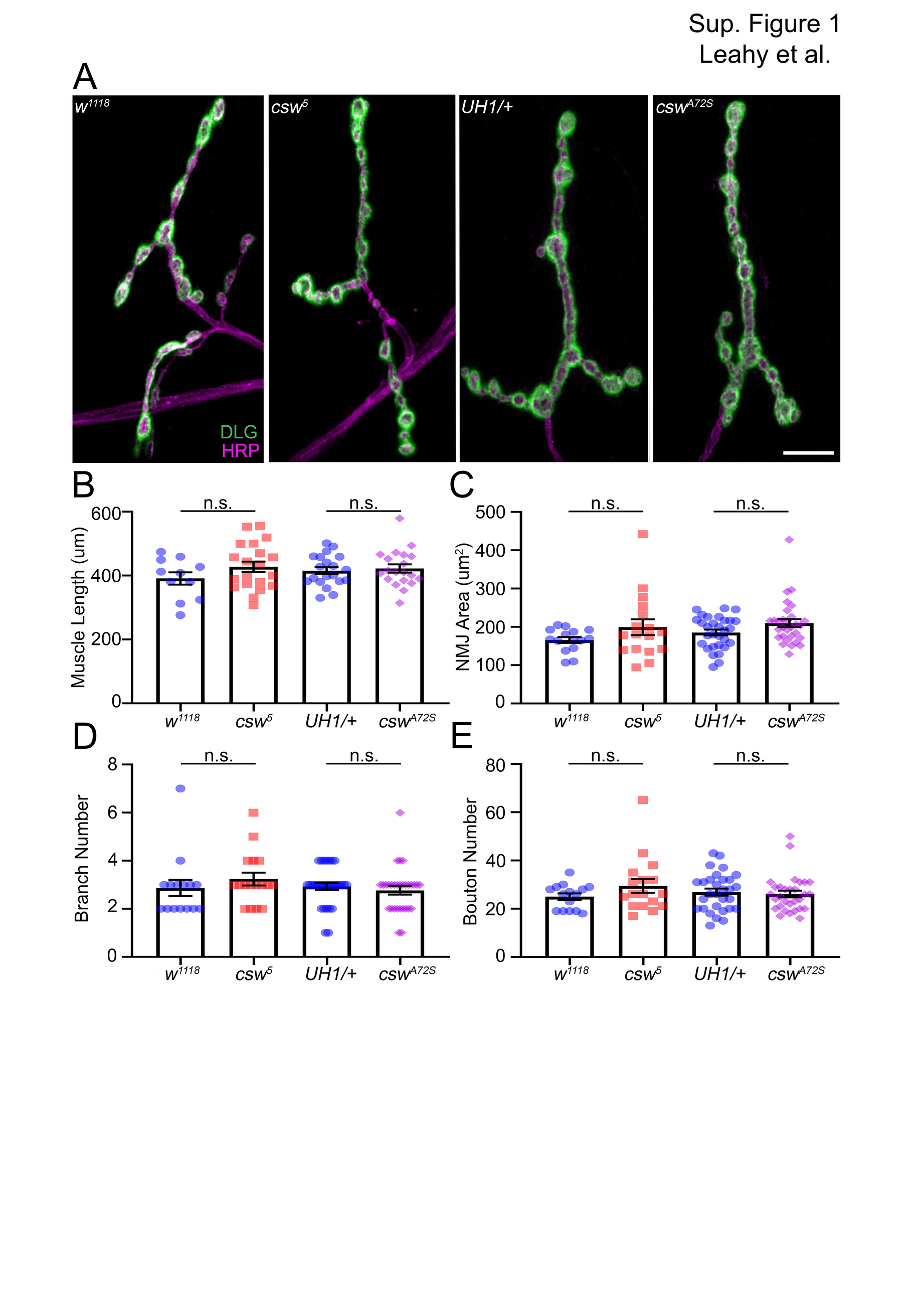

Supplement: S1 Fig — (A) Representative NMJ images of the w1118 genetic background control, csw5 null mutant, UH1-Gal4/w1118 transgenic driver control, and cswA72S GoF mutant (UH1-Gal4>cswA72S) colabeled for presynaptic membrane marker anti-HRP (magenta) and postsynaptic scaffold DLG (green). Scale bar: 10 μm. (B) Quantification of muscle length for all 4 genotypes using two-sided t tests. (C) Quantification of NMJ area for all 4 genotypes using Mann–Whitney tests. (D) Quantification of NMJ branch number for all 4 genotypes using Mann–Whitney tests. (E) Quantification of NMJ synaptic bouton number for all 4 genotypes using Mann–Whitney tests. Scatter plots show all the individual data points as well as mean ± SEM. N = number of NMJs. Significance: p > 0.05 (not significant, n.s.). The data underlying this figure can be found in S1 Data. csw, corkscrew; DLG, Discs Large; GoF, gain-of-function; HRP, horseradish peroxidase; NMJ, neuromuscular junction. (TIF) [file pbio.3001969.s001.tif]

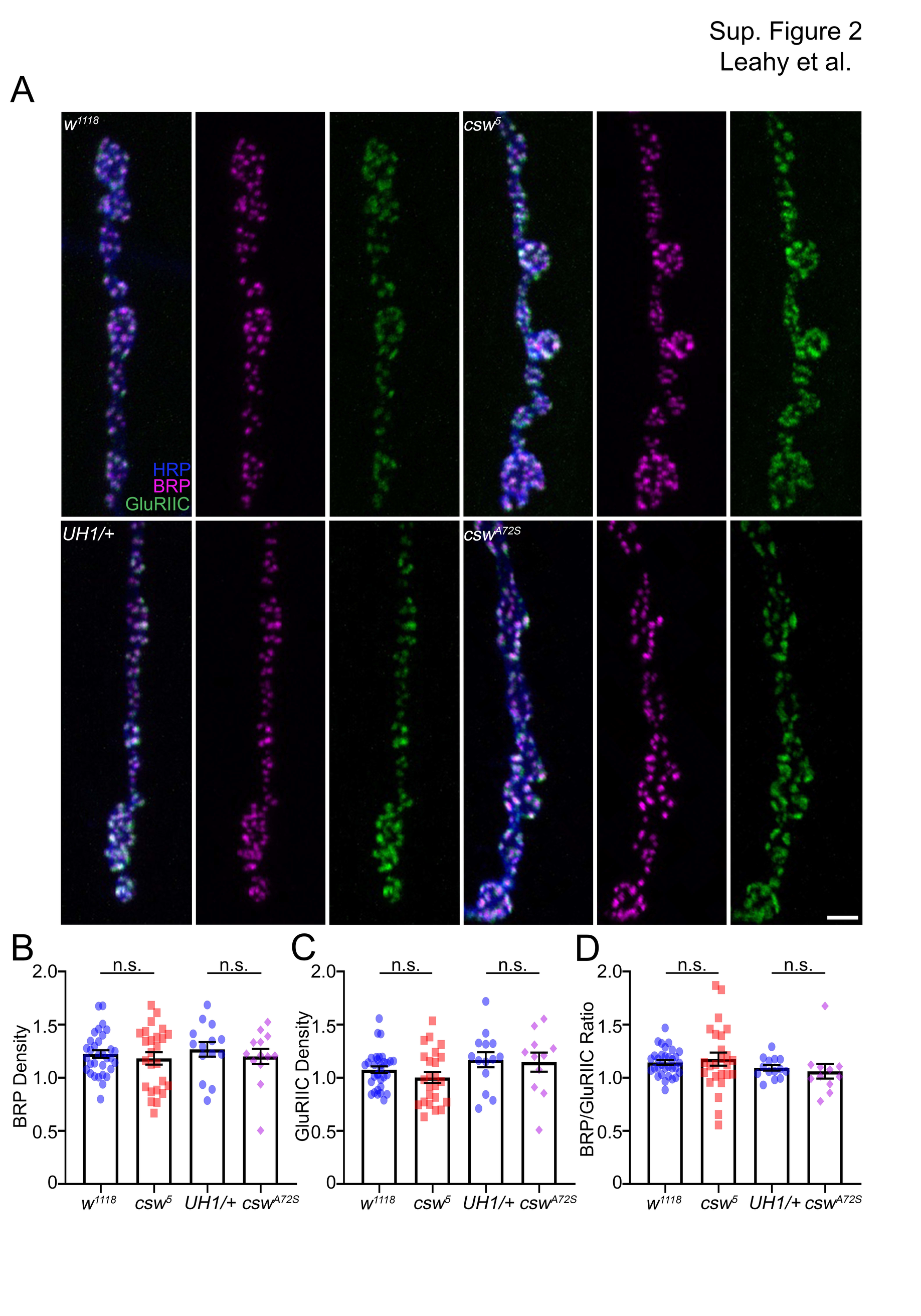

Supplement: S2 Fig — (A) Representative NMJ images of the w1118 genetic background control, csw5 null mutant, UH1-Gal4/w1118 transgenic driver control, and cswA72S GoF mutant (UH1-Gal4>cswA72S) colabeled for presynaptic membrane marker anti-HRP (blue), active zone marker Brp (magenta), and postsynaptic GluRIIC (green). Scale bar: 2.5 μm. (B) Quantification of Brp puncta density for all 4 genotypes using two-sided t test/Mann–Whitney tests. (C) Quantification of GluRIIC puncta density for all 4 genotypes using two-sided t tests. (D) Quantification of the Brp:GluRIIC puncta ratio for all 4 genotypes using two-sided t test/Mann–Whitney tests. Scatter plots show all the individual data points as well as mean ± SEM. N = number of NMJs. Significance: p > 0.05 (not significant, n.s.). The data underlying this figure can be found in S1 Data. Brp, Bruchpilot; csw, corkscrew; GluRIIC, glutamate receptor IIC; GoF, gain-of-function; HRP, horseradish peroxidase; NMJ, neuromuscular junction. (TIF) [file pbio.3001969.s002.tif]

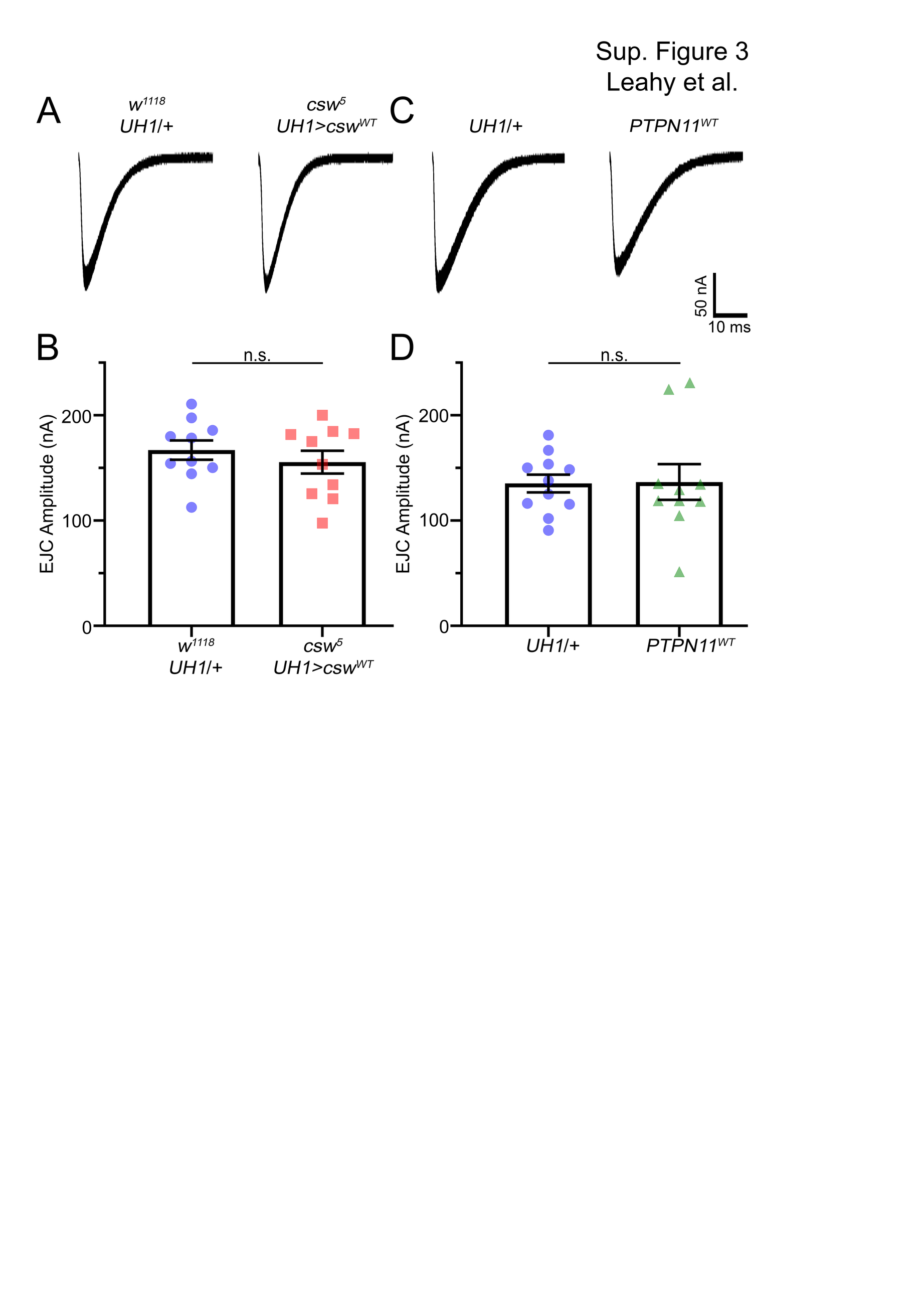

Supplement: S3 Fig — (A) Representative EJC traces for the csw5 null mutant rescued via expression of cswWT (csw5 UH1-Gal4>cswWT) and transgenic driver control (UH1-Gal4/w1118) showing 10 superimposed responses (1.0 mM Ca2). (B) Quantification of the mean EJC amplitudes using a two-sided t test. (C) Representative EJC traces for the wild-type PTPN11 (UH1-Gal4>PTPN11WT) and transgenic driver control (UH1-Gal4/w1118) showing 10 superimposed evoked synaptic responses (1.0 mM Ca2). (D) Quantification of the mean EJC amplitudes using a two-sided t test. Scatter plots show all the individual data points as well as mean ± SEM. N = number of NMJs. Significance: p > 0.05 (not significant, n.s.). The data underlying this figure can be found in S1 Data. Csw, corkscrew; EJC, excitatory junction current; NMJ, neuromuscular junction; PTPN11, protein tyrosine phosphatase non-receptor type 11. (TIF) [file pbio.3001969.s003.tif]

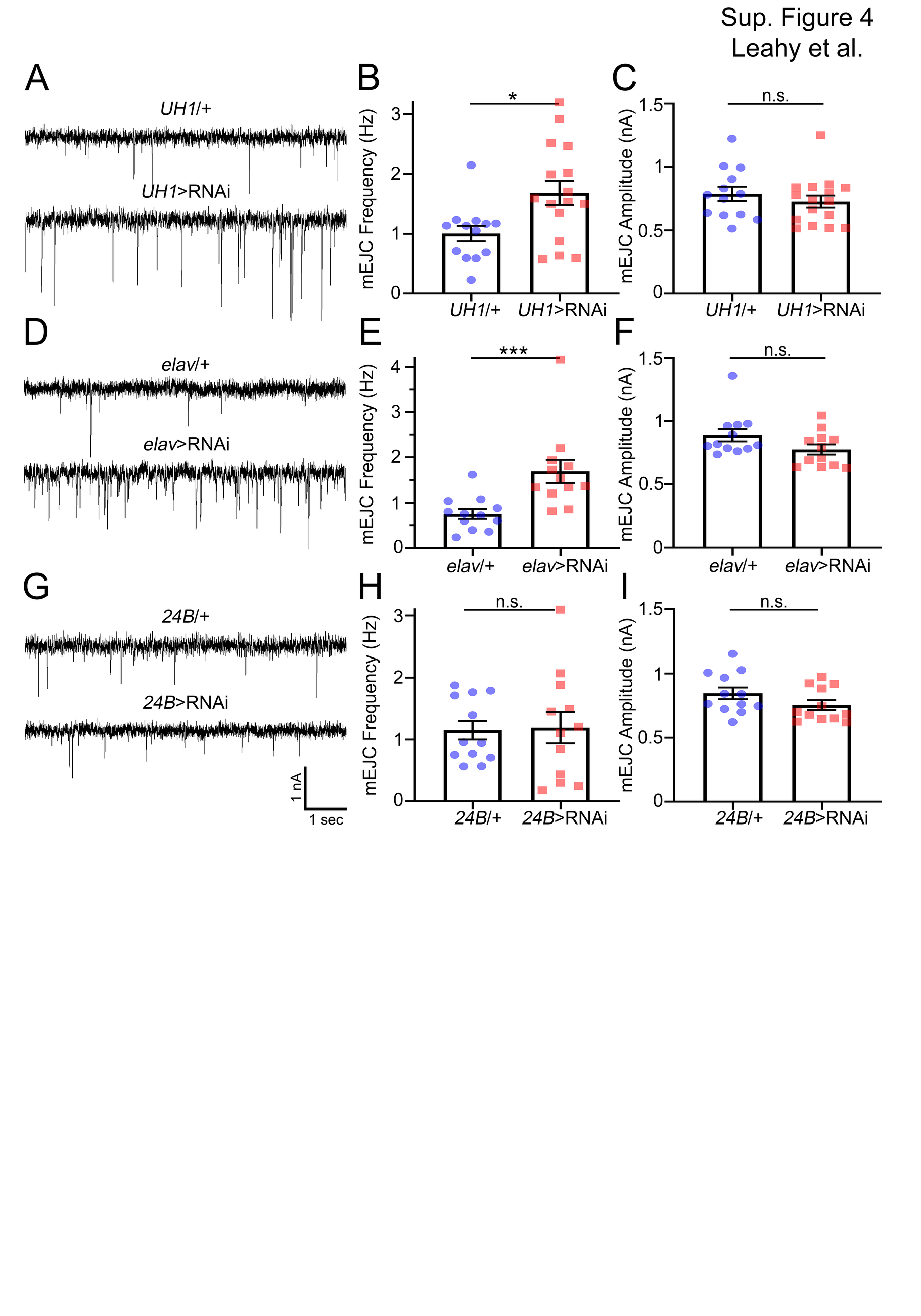

Supplement: S4 Fig — (A) Representative mEJC traces (1.0 mM Ca+2) in driver control (UH1-Gal4/TRiP control, top) and UH1-Gal4>csw RNAi (bottom). (B) Quantification of the mEJC frequency using a two-sided t test. (C) Quantification of mEJC amplitude using a Mann–Whitney test. (D) Representative mEJC traces (1.0 mM Ca+2) in driver control (elav-Gal4/TRiP control, top) and neuronal elav-Gal4>csw RNAi (bottom). (E) Quantification of the mEJC frequency using a Mann–Whitney test. (F) Quantification of the mEJC amplitude using a Mann–Whitney test. (G) Representative mEJC traces (1.0 mM Ca+2) in driver control (24B-Gal4/TRiP, top) and muscle 24B-Gal4>csw RNAi (bottom). (H) Quantification of the mEJC frequency using a two-sided t test. (I) Quantification of the mEJC amplitude using two-sided t test. Scatter plots show all the individual data points as well as mean ± SEM. N = number of NMJs. Significance: p > 0.05 (not significant, n.s.), p < 0.05 (*), and p > 0.001 (***). The data underlying this figure can be found in S1 Data. csw, corkscrew; mEJC, miniature EJC; NMJ, neuromuscular junction; RNAi, RNA interference. (TIF) [file pbio.3001969.s004.tif]

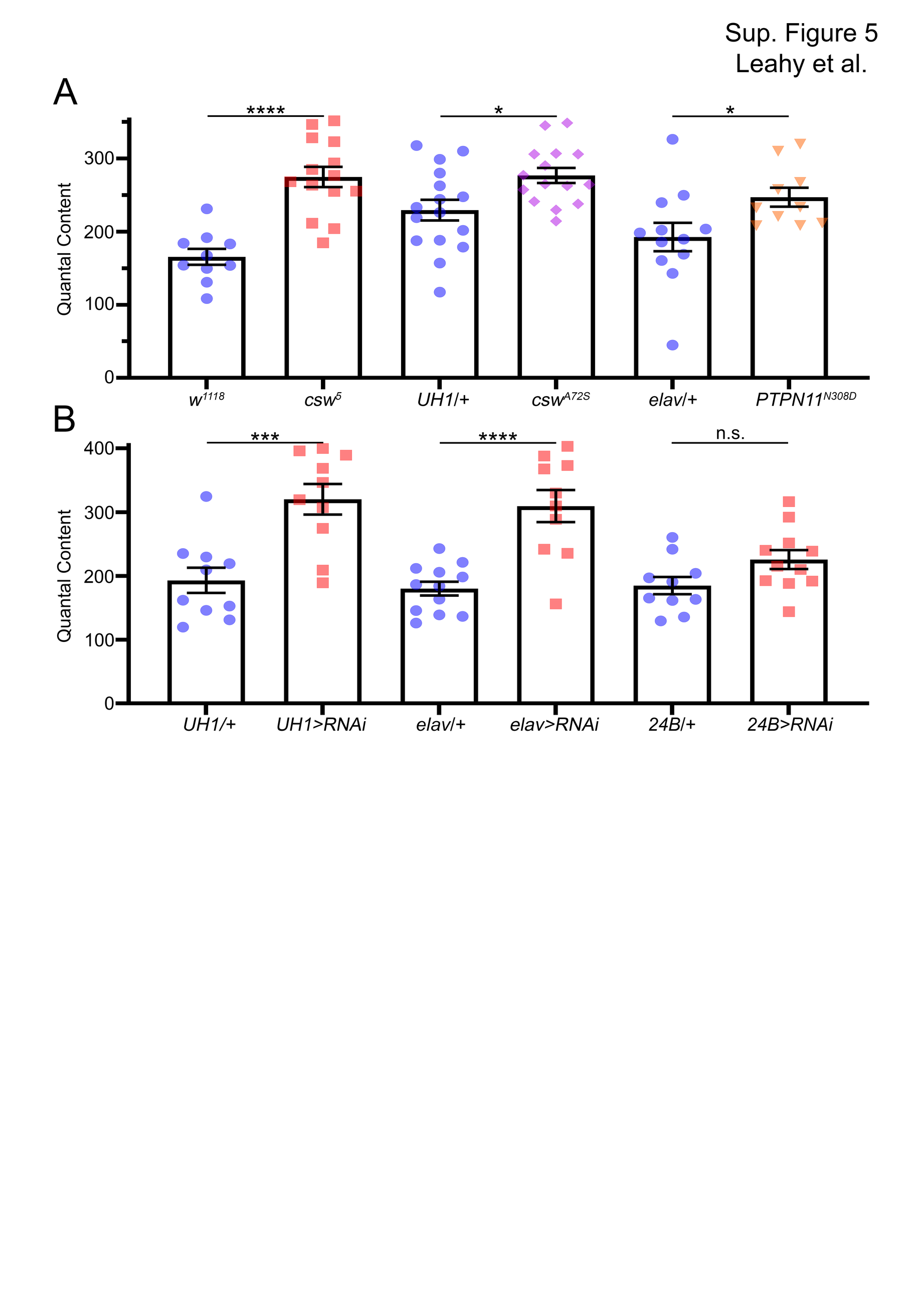

Supplement: S5 Fig — The quantal content at each NMJ was calculated by dividing the evoked EJC traces by the mean mEJC amplitude. (A) Quantification of the quantal content of both the csw/PTPN11 null and GoF mutants using two-sided t tests. (B) Quantification of the quantal content of csw RNAi ubiquitous (UH1), neuronal (elav), and muscle (24B) lines compared to their matched transgenic driver controls using two-sided t tests. Scatter plots show all the individual data points as well as mean ± SEM. N = number of NMJs. Significance: p > 0.05 (not significant, n.s.), p < 0.05 (*), p > 0.001 (***), and p < 0.0001 (****). The data underlying this figure can be found in S1 Data. Csw, corkscrew; EJC, excitatory junction current; GoF, gain-of-function; mEJC, miniature EJC; NMJ, neuromuscular junction; PTPN11, protein tyrosine phosphatase non-receptor type 11; RNAi, RNA interference. (TIF) [file pbio.3001969.s005.tif]

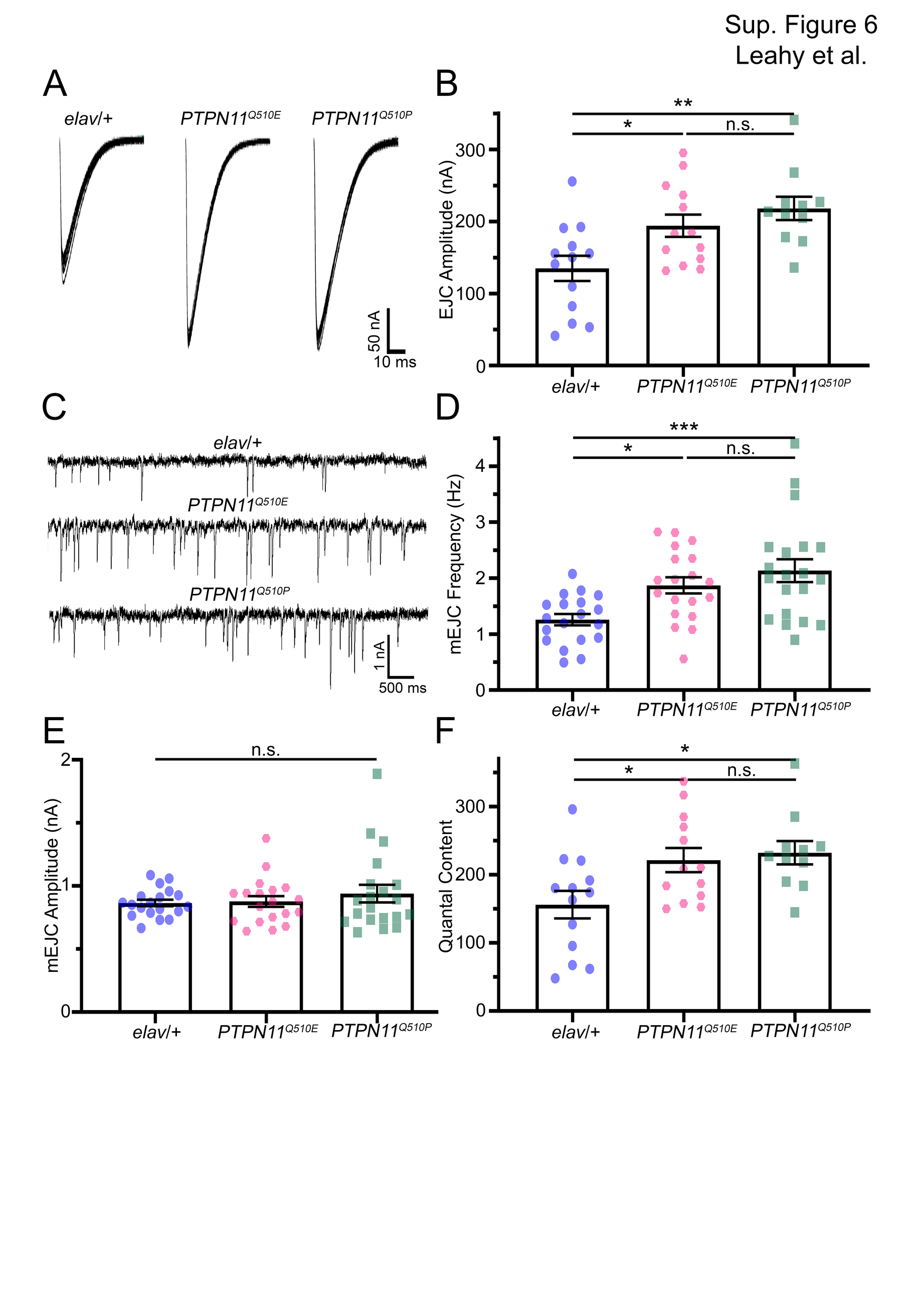

Supplement: S6 Fig — (A) Representative EJC traces for the transgenic driver control (elav-Gal4/w1118), and PTPN11 patient mutants PTPN11Q510E (elav-Gal4>PTPN11Q510E) and PTPN11Q510P (elav-Gal4>PTPN11Q510P) showing 10 superimposed evoked synaptic responses (1.0 mM Ca2+). (B) Quantification of the mean EJC amplitudes in all 3 genotypes using one-way ANOVA and Tukey’s multiple comparisons. (C) Representative mEJC traces (1.0 mM Ca2+) in above driver control (top), PTPN11Q510E (middle), and PTPN11Q510P (bottom). (D) Quantification of the mEJC frequency using one-way ANOVA and Tukey’s multiple comparisons. (E) Quantification of mEJC amplitude using a Kruskal–Wallis test. (F) Quantification of quantal content using one-way ANOVA and Tukey’s multiple comparisons. Scatter plots show all the individual data points as well as mean ± SEM. N = number of NMJs. Significance: p > 0.05 (not significant, n.s.), p < 0.05 (*), p < 0.001 (**), and p > 0.001 (***). The data underlying this figure can be found in S1 Data. EJC, excitatory junction current; mEJC, miniature EJC; NMJ, neuromuscular junction; NSML, NS with multiple lentigines; PTPN11, protein tyrosine phosphatase non-receptor type 11. (TIF) [file pbio.3001969.s006.tif]

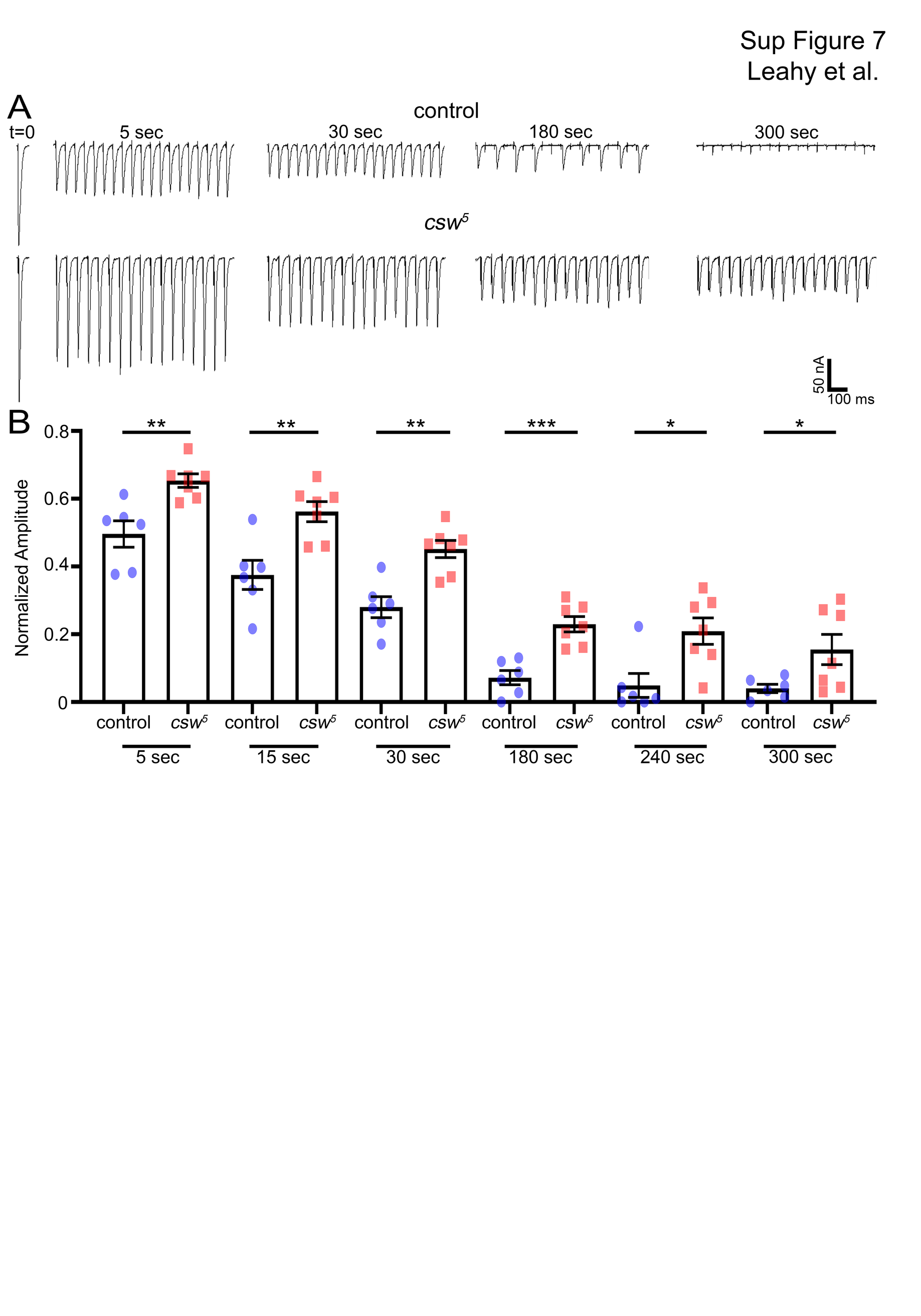

Supplement: S7 Fig — Prolonged HFS at 20 Hz (1 mM Ca+2) drives progressive synaptic amplitude depression over several minutes of continuous recording. (A) Representative evoked nerve-stimulated EJC traces at the basal frequency (t = 0) and indicated time points during the HFS train for the genetic background control (w1118, top) and the csw null mutant (csw5, bottom). (B) Quantification of normalized EJC amplitudes at the indicated time points during the HFS train using two-sided t tests. Scatter plots show all data points and mean ± SEM. N = number of NMJs. Significance: p < 0.05 (*), p < 0.001 (**), and p < 0.001 (***). The data underlying this figure can be found in S1 Data. csw, corkscrew; EJC, excitatory junction current; HFS, high-frequency stimulation; NMJ, neuromuscular junction. (TIF) [file pbio.3001969.s007.tif]

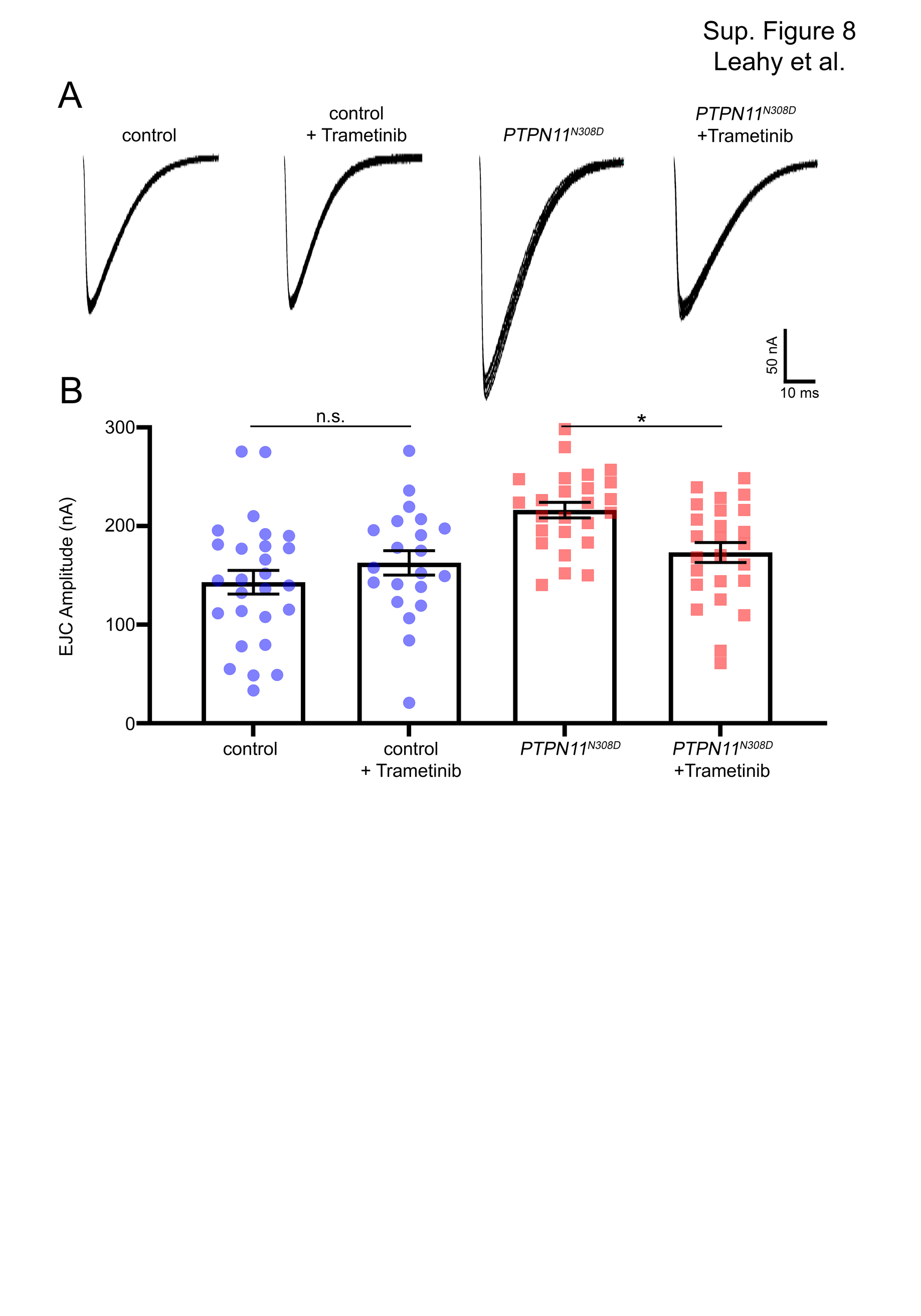

Supplement: S8 Fig — TEVC recordings with and without the pERK inhibiting drug Trametinib comparing the driver control (elav-Gal4/w1118) and NS GoF patient mutant (elav-Gal4>PTPN11N308D). (A) Representative EJC traces showing 10 superimposed responses (1.0 mM Ca+2) comparing the control (left) and PTPN11N308D mutant (right), with and without Trametinib. (B) Quantification of mean EJC amplitudes for all 4 conditions using one-way ANOVA and Tukey’s multiple comparisons. Scatter plots show all the data points and the mean ± SEM. N = number of NMJs. Significance: p > 0.05 (not significant, n.s.) and p < 0.05 (*). The data underlying this figure can be found in S1 Data. EJC, excitatory junction current; ERK, extracellular signal-regulated kinase; GoF, gain-of-function; NMJ, neuromuscular junction; NS, Noonan syndrome; pERK, phosphorylated ERK; PTPN11, protein tyrosine phosphatase non-receptor type 11; TEVC, two-electrode voltage-clamp. (TIF) [file pbio.3001969.s008.tif]

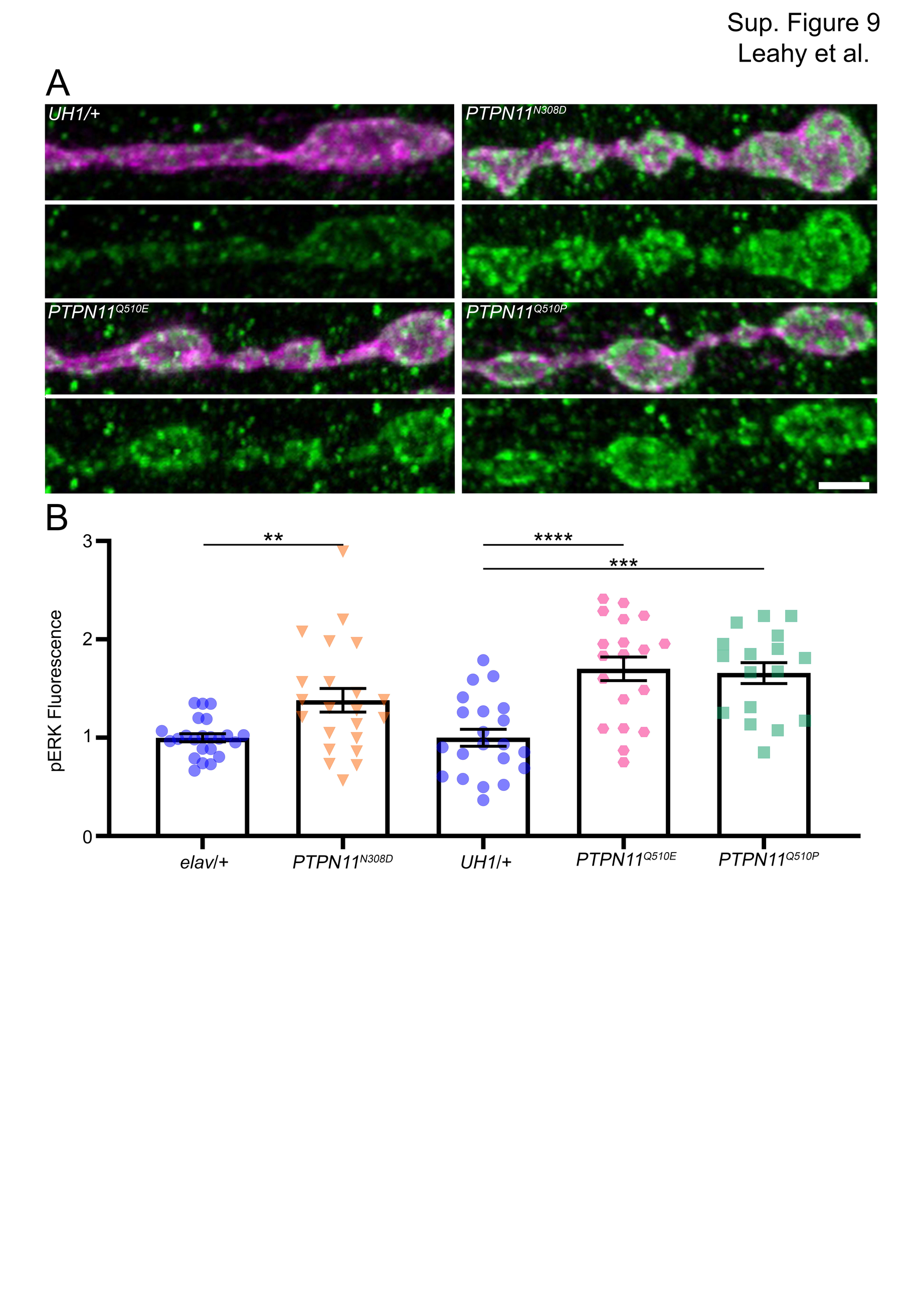

Supplement: S9 Fig — (A) Representative NMJ images of the driver control (UH1-Gal4/w1118, top left), the GoF mutant (elav>PTPN11N308D; top right), and two LoF mutants (UH1-Gal4>PTPN11Q510E, bottom left, and UH1-Gal4>PTPN11Q510P; bottom right) colabeled for presynaptic membrane marker anti-HRP (magenta) and pERK (green). Scale bar: 2.5 μm. (B) Quantified presynaptic anti-pERK fluorescence for all 5 genotypes using a two sided t test (PTPN11N308D) and one-way ANOVA and Tukey’s multiple comparisons (PTPN11Q510E/ PTPN11Q510P). Scatter plots show all data points and mean ± SEM. N = number of NMJs. Significance: p < 0.001 (**), p > 0.001 (***), and p < 0.0001 (****). The data underlying this figure can be found in S1 Data. GoF, gain-of-function; HRP, horseradish peroxidase; LoF, loss-of-function; NMJ, neuromuscular junction; pERK, phosphorylated ERK; PTPN11, protein tyrosine phosphatase non-receptor type 11. (TIF) [file pbio.3001969.s009.tif]

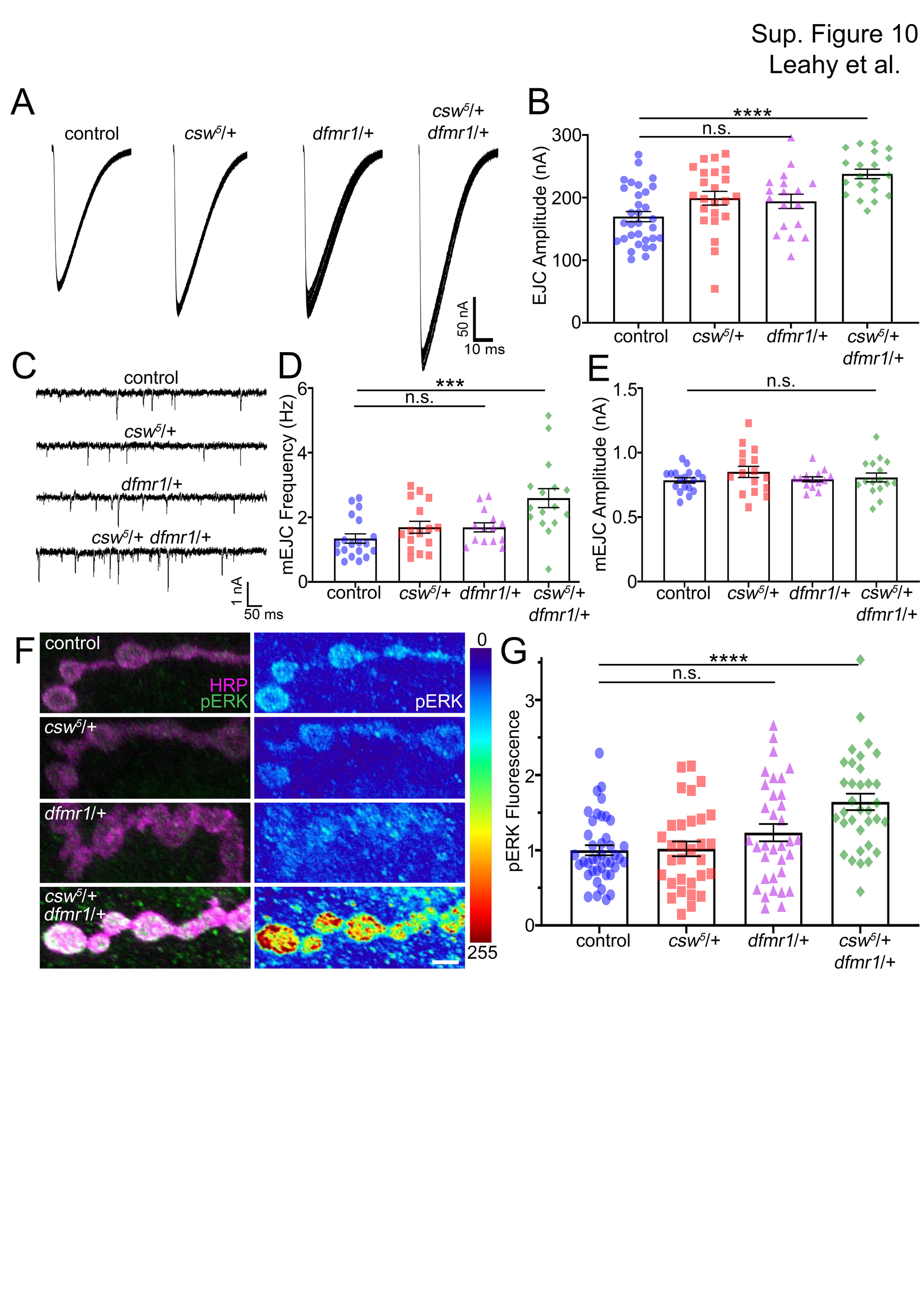

Supplement: S10 Fig — (A) Representative evoked EJC traces showing 10 superimposed TEVC recordings in background control (w1118), single heterozygotes (csw5/+ and dfmr150M/+), and the trans-heterozygote (csw5/+; dfmr150M/+). (B) Quantification of mean EJC amplitudes for all 4 genotypes using one-way ANOVA and Dunnett’s multiple comparisons. (C) Representative mEJC traces from the same 4 genotypes. (D) Quantification of mEJC frequency for all 4 genotypes using Kruskal–Wallis and Dunn’s multiple comparisons. (E) Quantification of mEJC amplitude for all 4 genotypes using Kruskal–Wallis. (F) Representative NMJ images from the same 4 genotypes colabeled for anti-pERK (green) and presynaptic membrane anti-HRP (magenta). pERK also shown as a heat map. Scale bar: 2.5 μm. (G) Quantification of normalized synaptic pERK fluorescence for all 4 genotypes using Kruskal–Wallis and Dunn’s multiple comparison tests. Scatter plots show all data points and the mean ± SEM. N = number of NMJs. Significance: p > 0.05 (not significant, n.s.), p < 0.001 (**), p > 0.001 (***), and p < 0.0001 (****). The data underlying this figure can be found in S1 Data. EJC, excitatory junction current; HRP, horseradish peroxidase; mEJC, miniature EJC; NMJ, neuromuscular junction; pERK, phosphorylated ERK; TEVC, two-electrode voltage-clamp. (TIF) [file pbio.3001969.s010.tif]

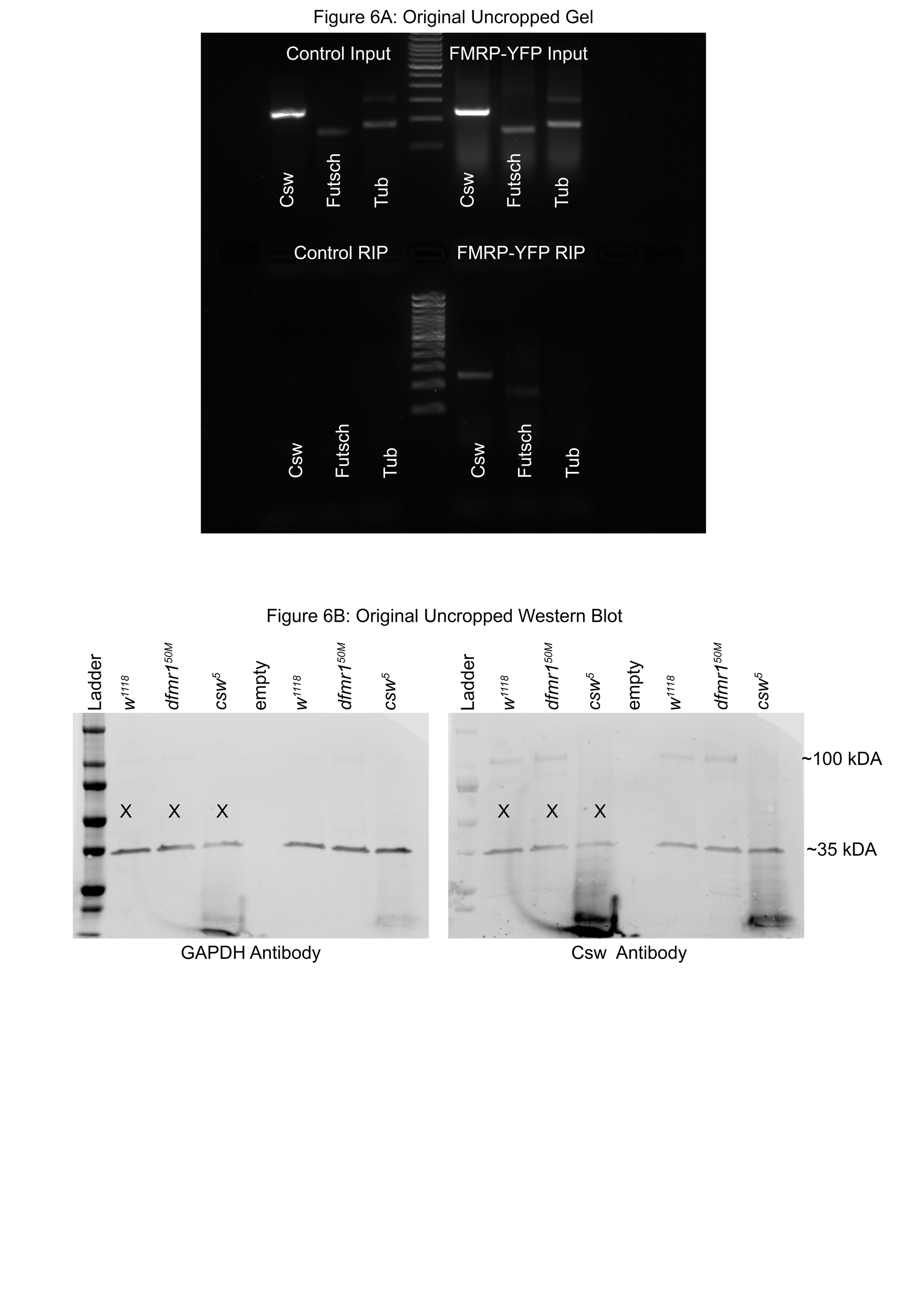

Supplement: S1 Raw Images — (TIF) [file pbio.3001969.s012.tif]
